# Supplementary material for: Vaccination with a structure-based stabilized version of malarial antigen Pfs48/45 elicits ultra-potent transmission-blocking antibody responses
Source: Immunity. 2022 Sep 13;55(9):1680–1692.e8. doi: 10.1016/j.immuni.2022.07.015 (PMC9487866; doi:10.1016/j.immuni.2022.07.015)
Supplement: Document S1. Figures S1–S6 and Tables S1–S10 [file mmc1.pdf]

Immunity, Volume *55*

Supplemental information

Vaccination with a structure-based stabilized version of malarial antigen Pfs48/45 elicits ultra-potent transmission-blocking antibody responses

Brandon McLeod, Moustafa T. Mabrouk, Kazutoyo Miura, Rashmi Ravichandran, Sally Kephart, Sophia Hailemariam, Thao P. Pham, Anthony Semesi, Iga Kucharska, Prasun Kundu, Wei-Chiao Huang, Max Johnson, Alyssa Blackstone, Deleah Pettie, Michael Murphy, John C. Kraft, Elizabeth M. Leaf, Yang Jiao, Marga van de Vegte-Bolmer, Geert-Jan van Gemert, Jordache Ramjith, C. Richter King, Randall S. MacGill, Yimin Wu, Kelly K. Lee, Matthijs M. Jore, Neil P. King, Jonathan F. Lovell, and Jean-Philippe Julien

Supplementary Material

Fig S1:

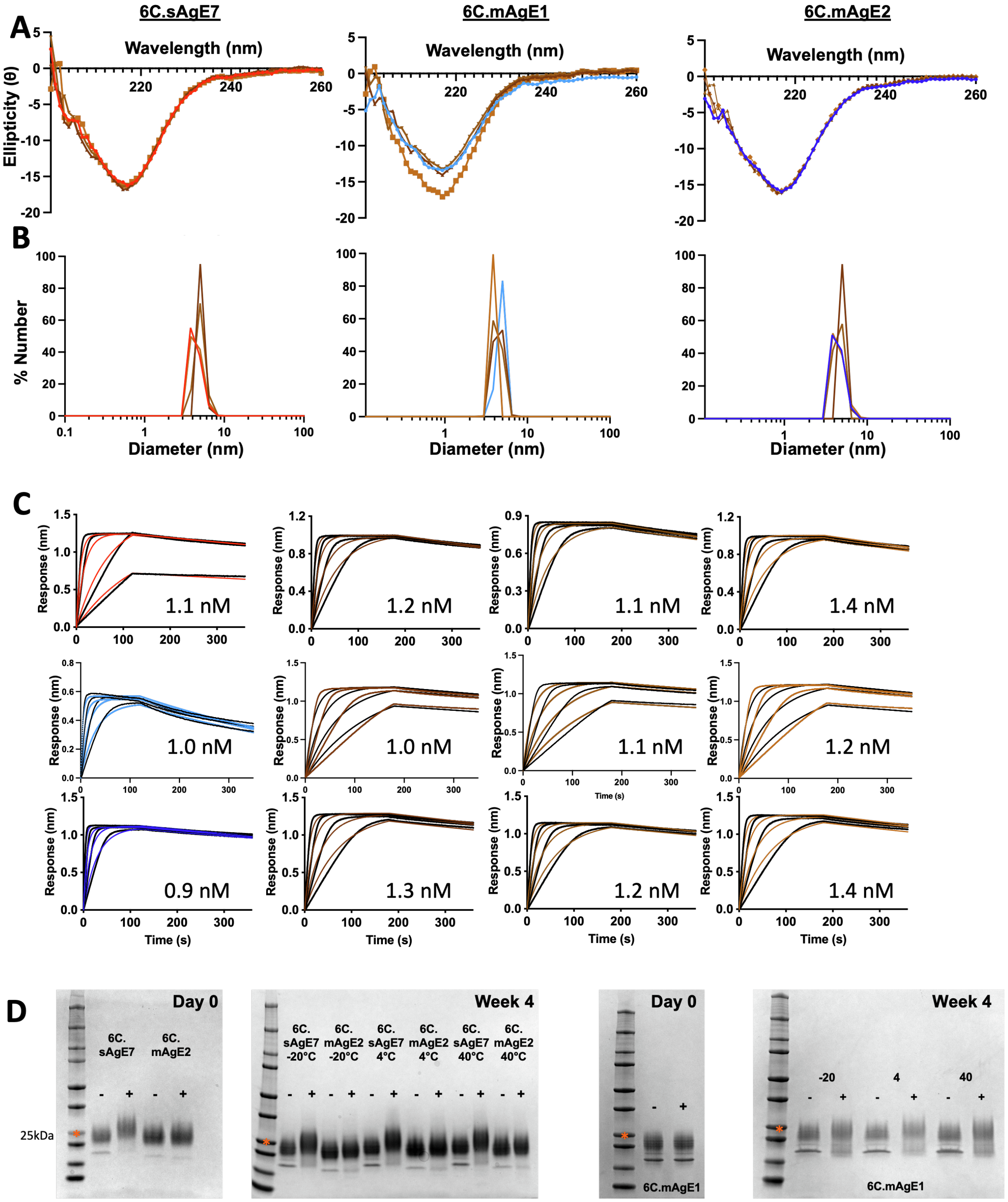

Fig S1. Accelerated thermostability assessment of 6C.sAgE7, 6C.mAgE1, and 6C.mAgE2 at Day 0 and Week 4. A) Day 0 CD wavelength scans of 6C.sAgE7 (left, red), 6C.mAgE1 (mid, pale blue), and 6C.mAgE2 (right, blue), superimposed with full CD wavelength scans from samples incubated for four weeks at -20°C (dark brown), 4°C (brown), and 40°C (light brown). **B)** Superimposition of DLS for stabilized antigens at Day 0 and Week 4, colored as in **A**. **C)** Day 0 and Week 4 representative binding curves used to calculate binding constants for 6C.sAgE7 (top), 6C.mAgE1 (middle) and 6C.mAgE2 (bottom). Affinity constant (KD) is labelled on its respective binding curve for raw data (black) and fitted data (as colored in **A**), for four serially diluted concentrations of Pfs48/45-6C antigen, from 250 - 31.3 nM) **A**. **D)** SDS-PAGE of Day 0 and Week4 samples. Symbols "-" and "+" denote which samples were run without and with reducing agent, respectively. Relevant 25 kDa band from molecular weight ladder is denoted by an orange asterisk. (related to **Fig 1**)

Fig S2:

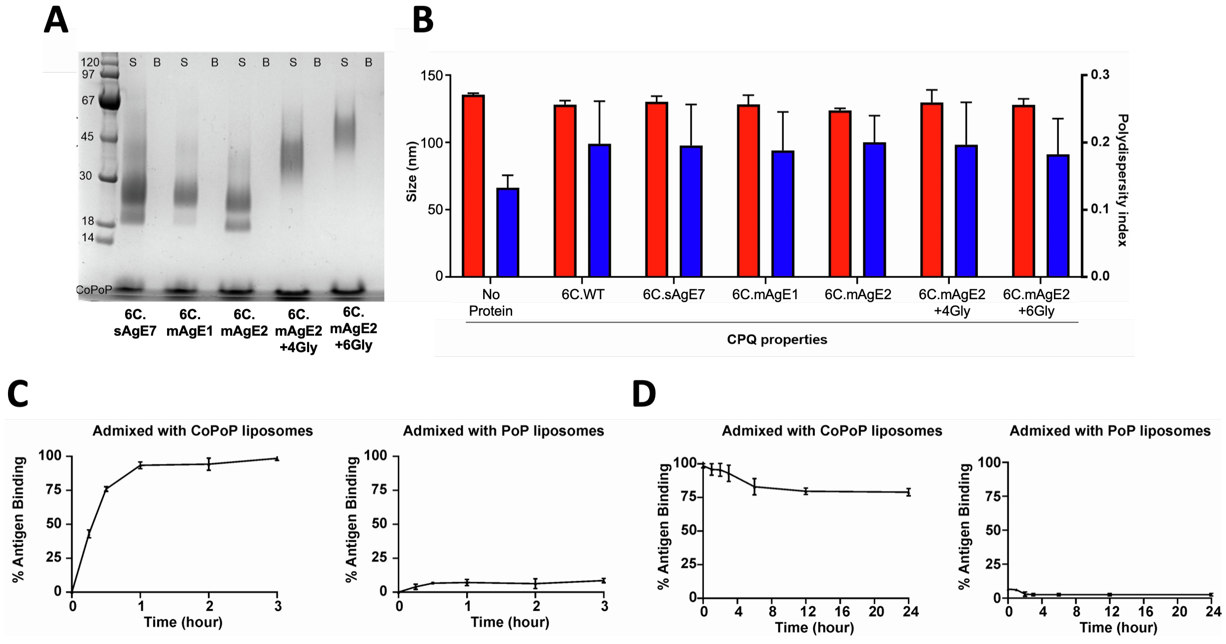

Fig S2. Particle properties of CPQ liposomes with Pfs48/45-6C variants. A) Nickel-NTA bead competition assay. Proteins were incubated with CPQ liposomes, then subjected to competition with Nickel-NTA beads. Beads were separated into the supernatant (S) and beads (B) fractions. Protein presence in the supernatant fraction reflects full binding of the protein to the liposomes. **B)** Size (red, left Y-axis) and polydispersity (blue, right Y-axis) of liposomes displaying the various indicated Pfs48/45-6C constructs plotted as mean, with error as standard deviation. **C)** Fluorescently labeled Pfs48/45-6C was incubated with CPQ liposomes or similar liposomes lacking cobalt within the porphyrin-phospholipid (PoP) as indicated to determine binding kinetics in PBS. Upon binding to liposomes, energy transfer induces a decrease in fluorescence reflecting antigen binding. Data is plotted as mean, with standard deviation as error. **D)** Following complexation, stability in 20% serum over time at 37 °C. Data is plotted as mean, with standard deviation as error. (related to **Fig 2**)

Fig S3:

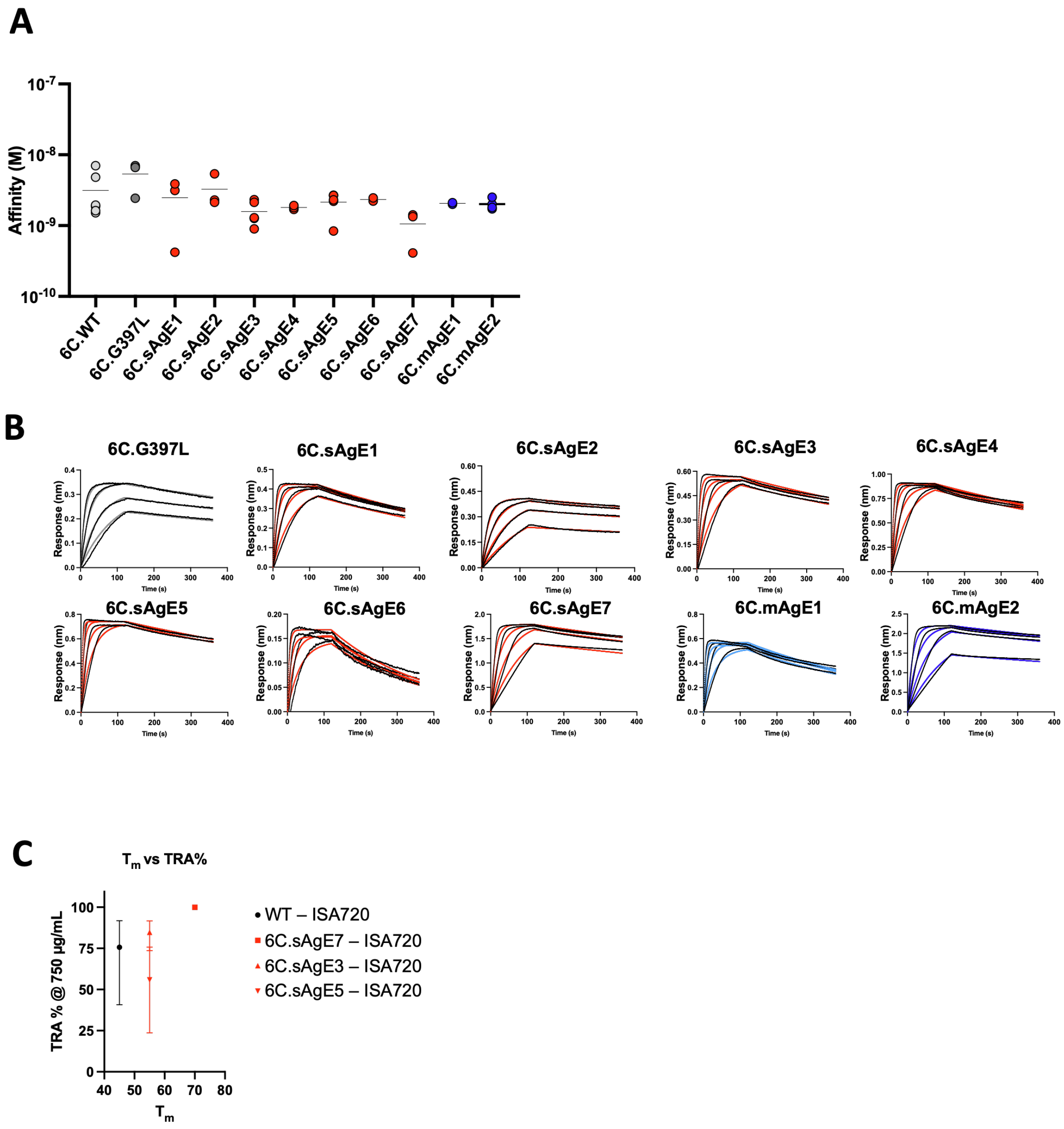

Fig S3. Biophysical characterization of Pfs48/45-6C stability mutations. A) Affinity of TB31F Fab binding to Pfs48/45-6C variants as measured by BLI. Symbols are representative of individual measurements, with black horizontal bar indicating the mean. **B)** Representative curves of

TB31F Fab binding to Pfs48/45-6C variants as measured by BLI. Curves are presented as raw data (black), and fitted data (as coloured in **A**), for four serially diluted concentrations of 6C antigen, from 250 - 31.3 nM) **C)** SMFA for IgG purified from mice immunized with ISA720-adjuvanted antigens were tested at 750 μg/mL, and plotted as a function of thermostability (T_m). Symbol represents the best-estimate TRA(%) of the two independent feeds, and error bars are 95% CI. (related to **Figs 1-3**)

Fig S4:

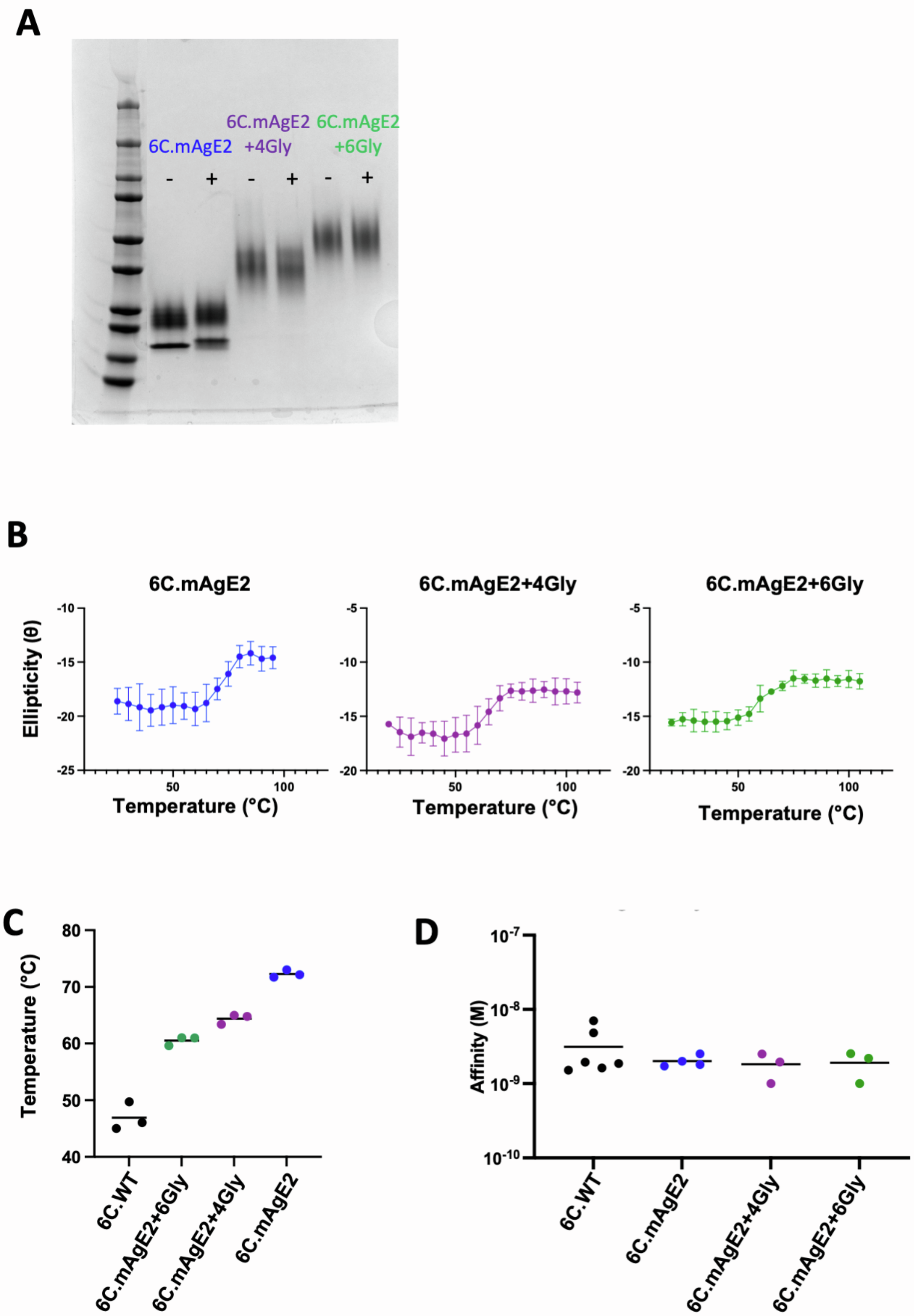
A
6C.mAgE2
6C.mAgE2 +4Gly
6C.mAgE2 +6Gly
- + - + - +
B
6C.mAgE2
6C.mAgE2+4Gly
6C.mAgE2+6Gly
Ellipticity (θ)
Temperature (°C)
C
Temperature (°C)
6C.WT
6C.mAgE2+6Gly
6C.mAgE2+4Gly
6C.mAgE2
D
Affinity (M)
6C.WT
6C.mAgE2
6C.mAgE2+4Gly
6C.mAgE2+6Gly

Fig S4. Characterization of hyperglycosylated Pfs48/45-6C variants. A) SDS-PAGE of 6C.mAgE2 hyperglycosylated variants in comparison to 6C.mAgE2. **B)** CD melting curves for 6C.mAgE2 and hyperglycosylated variants. Data points represent mean of three independent replicates and error bars are plotted as standard deviation. **C)** Thermostability of 6C.WT, 6C.mAgE2 and hyperglycosylated variants, as determined by CD melting curves in **B**. Data is plotted with individual replicates, with mean as black bar. **D)** Affinity measurements of TB31F Fab to 6C.WT, 6C.mAgE2 and hyperglycosylated variants via BLI. Data are plotted as independent replicates, with the black bar representing the mean. (related to **Fig 4**)

Fig S5:

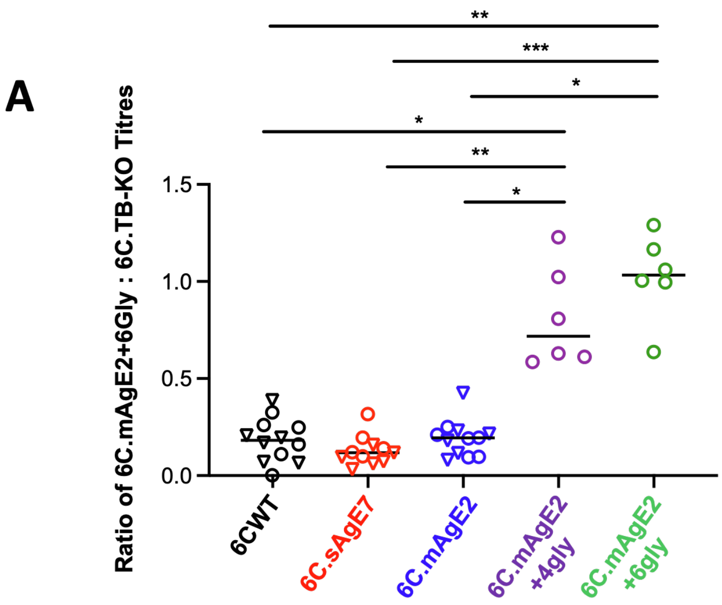

Fig S5. Characterization of immune response elicited by hyperglycosylated Pfs48/45-6C variants. Ratio of ELISA titres derived by dividing reactogenicity to 6C.mAgE2+6gly over reactogenicity to a Pfs48/45-6C TB31F epitope-knockout construct. Data is plotted as responses from individual mice, with the horizontal black bar representing the mean. *, **, and *** indicate p values of ≤0.05, ≤0.01, and ≤0.001, respectively as determined by one-way ANOVA. (related to **Fig 4**)

Fig S6:

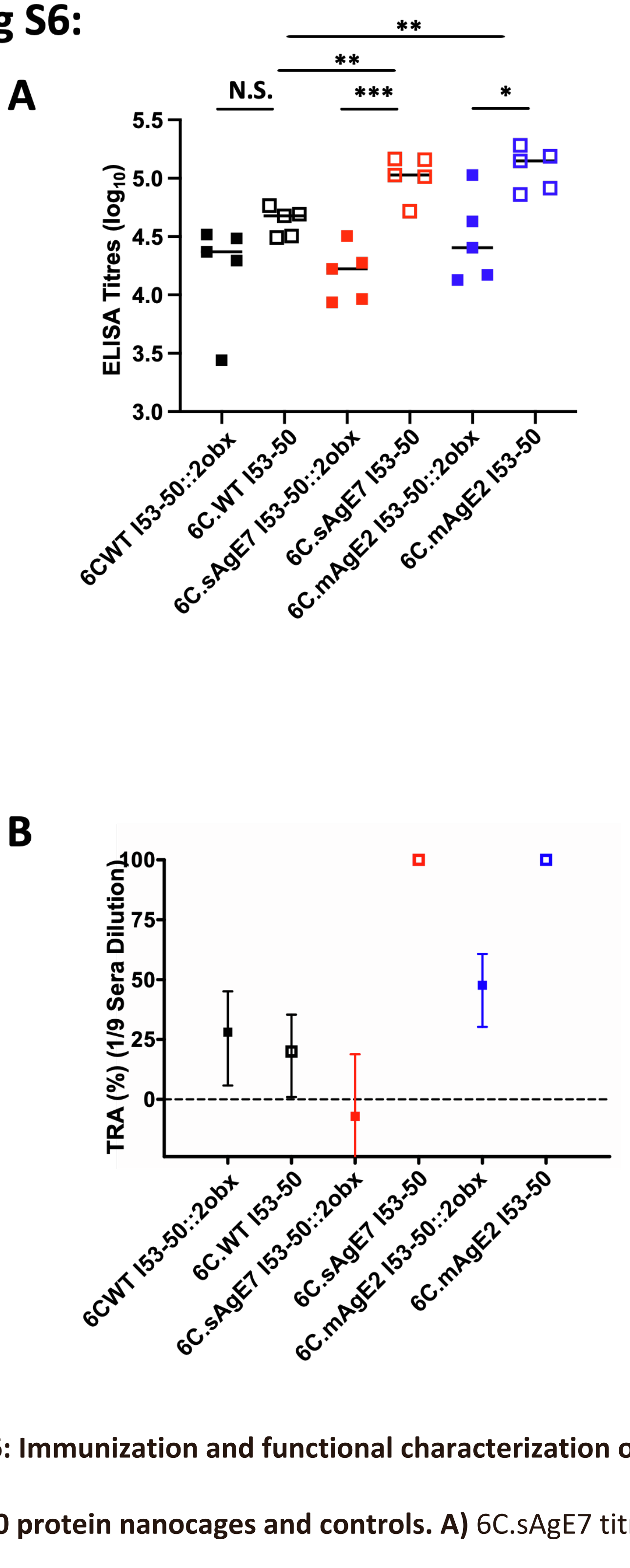

Fig S6: Immunization and functional characterization of Pfs48/45-6C stabilized variants on I53-50 protein nanocages and controls. A) 6C.sAgE7 titres elicited by stabilized Pfs48/45-6C variants, as non-assembling mixtures of Pfs48/45-6C-I53-50A trimers and 2obx pentamers

(filled squares) or icosahedral I53-50 protein nanocages (open squares), measured by ELISA. Data is plotted as individual mice, with the horizontal black bars representing the mean. n.s. non-significant ($p>0.05$); *, **, and *** indicate p values of ≤0.05, ≤0.01, and ≤0.001, respectively as determined by one-way ANOVA for log-transformed ELISA titres. **B)** SMFA with 1:9 diluted pooled sera derived from mice immunized with stabilized Pfs48/45-6C variants at a 1:9 dilution for non-assembling controls (filled circles) or icosahedral particles (open circles), plotted as mean with error as standard deviation. (related to **Fig 5**)

Supplementary table 1: Data collection and refinement statistics for the 6C.mAgE1-RUPA-47 Fab-RUPA-117 Fab complex. (related to **Fig 3**)

	6C.mAgE1-RUPA-47 Fab-RUPA-117 Fab
**Wavelength (Å)**	1.033190
**Space group**	$P2_12_12_1$
**Cell dimensions**	
a, b, c (Å)	73.7 127.2 132.7
α, β, γ **(°)**	90 90 90
**Resolution (Å)**	29.43 - 2.18 (2.21 - 2.18)
**No. molecules in ASU**	1
**No. unique observations**	65,804 (2596)
**Multiplicity**	12.6 (7.8)
**R_{merge} (%)**	20.5 (73.3)
**R_{pim} (%)**	5.9 (28.1)
**<I/sigma I>**	8.7 (1.5)
**$CC_{1/2}$**	99.5 (60.3)
**Completeness (%)**	99.9 (100)
**Refinement Statistics**	
**Non-hydrogen atoms**	8,338
**Macromolecule**	7,730
**Water**	594
**Hetero atom**	14
**R_{factor}/ R_{free}**	17.8/ 22.8
**Rms deviations from ideality**	
**Bond lengths (Å)**	0.003
**Bond angle (°)**	0.66
**Ramachandran plot**	
**Favoured regions (%)**	97.5
**Allowed regions (%)**	2.5
**Outliers (%)**	0.1
**B-factors (Å^2)**	
**Wilson B-factor**	31.0
**Average B-factors**	33.2
**Average macromolecule**	32.9
**Average hetero atom**	63.3
**Average water**	36.4

Supplementary table 2: Conditions for HDX-MS experiment. (related to **Fig 3**)

Dataset	6C.sAgE7	6C.mAgE2
HDX reaction details	85% D2O buffer, pH* 7.659, labeled at RT. Quenched at pH 2.55 in 200mM TCEP, 0.2%FA	85% D2O buffer, pH* 7.659, labeled at RT. Quenched at pH 2.55 in 200mM TCEP, 0.2%FA
HDX time course	3 s, 1 min, 30 min, 5 h	3 s, 1 min, 30 min, 5 h
HDX controls	PPPI, PPPF	PPPI, PPPF
Back-exchange	37.1 +/- 7.8%	37.0 +/- 8.7%
Number of peptides	32	39
Sequence coverage	87%	88%
Average peptide length/redundancy	11.4 residue length/2.8 redundancy	11.3 residue length/ 3.25 redundancy
Replicates (biological or technical)	two technical replicates	two technical replicates
Repeatability	stddev of 1.2% across technical replicates	stddev of 1.2% across technical replicates

Supplementary Table 3: SMFA data for Fig 2C and 2D

Feed #	Animal study #	Immunogen	Adjuvant	IgG Conc. [µg/mL]	Mean oocyst	TRA (%)	p-value[a]
#296	*Control IgG*			*750*	*66*		
	UB19	6C.sAgE3	CPQ	750	42	37	0.241
	UB19	6C.sAgE3	ISA	750	22	67	0.005
	UB19	6C.sAgE5	CPQ	750	42	36	0.277
	UB19	6C.sAgE5	ISA	750	27	60	0.018
	UB19	6C.sAgE7	CPQ	750	0	100	0.001
	UB19	6C.sAgE7	ISA	750	0	100	0.001
#304	*Control IgG*			*750*	*57*		
	UB19	6C.sAgE7	CPQ	750	0	100	0.001
	UB19	6C.sAgE7	ISA	750	0	100	0.001
#312	*Control IgG*			*750*	*15*		
	UB21	6C.WT	CPQ	750	3	78	0.002
	UB21	6C.WT	ISA	750	4	76	0.003
#321	*Control IgG*			*750*	*16*		
	UB19	6C.sAgE3	CPQ	750	6	64	0.019
	UB19	6C.sAgE3	ISA	750	1	93	0.001
	UB19	6C.sAgE5	CPQ	750	5	70	0.005
	UB19	6C.sAgE5	ISA	750	8	52	0.089

[a] The p-values were calculated using a zero-inflated negative binomial (ZINB) model (Miura *et al.*, 2016).

Supplementary Table 4: SMFA data for Fig 2E

Feed #	Animal study #	Immunogen	Adjuvant	Anti-6C.sAgE7 ELISA units	Mean oocyst	TRA (%)	p-value
#296	*Control IgG*				*66*		
	UB19	6C.sAgE7	CPQ	21,455	0	100	0.001
	UB19	6C.sAgE7	ISA	7,410	0	100	0.001
#304	*Control IgG*				*57*		
	UB19	6C.sAgE7	CPQ	21,455	0	100	0.001
	UB19	6C.sAgE7	ISA	7,410	0	100	0.001
#312	*Control IgG*				*15*		
	UB21	6C.sAgE7	CPQ	21,455	0	100	0.001
	UB21	6C.sAgE7	ISA	7,410	0	100	0.001
#315	*Control IgG*				*54*		
	UB21	6C.sAgE7	CPQ	1,345	8	85	0.001
				2,689	1	97	0.001
				5,378	0	100	0.001
				10,728	0	100	0.001
	UB21	6C.sAgE7	ISA	464	39	28	0.389
				929	47	12	0.726
				1,857	26	52	0.078
				3,705	5	92	0.001
#317	*Control IgG*				*14*		
	UB21	6C.sAgE7	CPQ	2,689	0	99	0.001
				5,378	0	100	0.001
				10,728	0	100	0.001
#321	*Control IgG*				16		
	UB21	6C.sAgE7	CPQ	671	5	65	0.015
				1,341	2	90	0.001
#325	*Control IgG*				*9*		
	UB21	6C.sAgE7	CPQ	671	8	9	0.799

Supplementary Table 5: SMFA data for Fig 4C

Feed #	Animal study #	Immunogen	Adjuvant	IgG Conc. [μg/mL]	Mean oocyst	TRA (%)	p-value
#317	*Control IgG*				*14*		
	UB22	6C.mAgE2	CPQ	375	0	100	0.001
#318	*Control IgG*				*6*		
	UB22	6C.mAgE2	CPQ	375	0	100	0.001
	UB22	6C.mAgE2+4gly	CPQ	375	1	90	0.001
	UB22	6C.mAgE2+6gly	CPQ	375	9	-38	0.423
#321	*Control IgG*				*16*		
	UB22	6C.mAgE2+4gly	CPQ	375	3	83	0.001
	UB22	6C.mAgE2+6gly	CPQ	375	4	72	0.004

Supplementary Table 6: SMFA data for Fig 5C

Feed #	Animal study #	Immunogen	Adjuvant	IgG Conc. [μg/mL]	Mean oocyst	TRA (%)	p-value
#296	*Control IgG*				*66*		
	UB19	6C.sAgE7	CPQ	750	0	100	0.001
#304	*Control IgG*				*57*		
	UB19	6C.sAgE7	CPQ	750	0	100	0.001
#312	*Control IgG*				*15*		
	UB21	6C.WT	CPQ	750	3	78	0.002
	UB21	6C.sAgE7	CPQ	750	0	100	0.001
	UB21	6C.mAgE1	CPQ	750	0	100	0.001
	UB21	6C.mAgE2	CPQ	750	0	100	0.001
#315	*Control IgG*				*54*		
	UB21	6C.sAgE7	CPQ	47	8	85	0.001
			CPQ	94	1	97	0.001
			CPQ	188	0	100	0.001
			CPQ	375	0	100	0.001
	UB21	6C.mAgE2	CPQ	47	34	38	0.224
			CPQ	94	22	59	0.036
			CPQ	188	5	91	0.001
			CPQ	375	0	99	0.001
#317	*Control IgG*				*14*		
	UB21	6C.mAgE1	CPQ	94	0	100	0.001
			CPQ	188	0	100	0.001
			CPQ	375	0	100	0.001
	UB21	6C.mAgE2	CPQ	94	10	25	0.479
			CPQ	188	1	92	0.001
			CPQ	375	0	100	0.001
	UB21	6C.sAgE7	CPQ	94	0	99	0.001
				188	0	100	0.001
				375	0	100	0.001
	UB22	6C.mAgE2	CPQ	94	1	96	0.001
				188	0	100	0.001
				375	0	100	0.001
	UB22	6C.sAgE7	CPQ	94	0	100	0.001
			CPQ	188	0	100	0.001
			CPQ	375	0	100	0.001
#318	*Control IgG*				*6*		
	UB22	6C.WT	CPQ	375	4	34	0.287
	UB22	6C.sAgE7	CPQ	375	0	100	0.001
	UB22	6C.mAgE2	CPQ	375	0	100	0.001

Supplementary Table 7: SMFA data for Fig 5C (continued)

Feed #	Animal study #	Immunogen	Adjuvant	IgG Conc. [μg/mL]	Mean oocyst	TRA (%)	p-value
#321	*Control IgG*				*16*		
	UB21	6C.mAgE1	CPQ	23	4	72	0.004
			CPQ	47	3	81	0.001
			CPQ	94	1	91	0.001
	UB21	6C.sAgE7	CPQ	23	5	65	0.015
			CPQ	47	2	90	0.001
	UB22	6C.mAgE2	CPQ	23	2	86	0.001
			CPQ	23	7	56	0.049
			CPQ	47	4	76	0.001
			CPQ	94	2	89	0.001
			CPQ	188	1	96	0.001
	UB22	6C.sAgE7	CPQ	47	1	91	0.001
	UB21	6C.mAgE1	CPQ	23	4	72	0.004
			CPQ	47	3	81	0.001
#325	*Control IgG*				*9*		
	UB21	6C.mAgE1	CPQ	23	5	48	0.119
			CPQ	47	0	95	0.001
			CPQ	94	0	100	0.001
			CPQ	188	0	100	0.001
			CPQ	375	0	100	0.001
	UB21	6C.sAgE7	CPQ	23	8	9	0.799
	UB22	6C.mAgE2	CPQ	23	6	37	0.276
			CPQ	47	3	69	0.015
	UB22	6C.sAgE7	CPQ	23	6	37	0.228
			CPQ	47	3	64	0.014
			CPQ	94	1	94	0.001
			CPQ	188	0	100	0.001
	UB22	6C.WT	CPQ	375	4	73	0.006
#332	*Control IgG*				*100*		
	UB21	6C.WT	CPQ	375	47	53	0.051
				750	52	48	0.093
	UB22	6C.WT	CPQ	750	65	35	0.264

Supplementary Table 8: SMFA data for Fig S3C

Feed #	Animal study #	Immunogen	Adjuvant	IgG Conc. [μg/mL]	Mean oocyst	TRA (%)	p-value
#296	*Control IgG*				*66*		
	UB19	6C.sAgE3	CPQ	750	22	67	0.005
	UB19	6C.sAgE5		750	27	60	0.018
	UB19	6C.sAgE7		750	0	100	0.001
#304	*Control IgG*				*57*		
	UB19	6C.sAgE7	CPQ	750	0	100	0.001
#321	*Control IgG*				*16*		
	UB19	6C.sAgE3	CPQ	750	1	93	0.001
	UB19	6C.sAgE5	CPQ	750	8	52	0.089

Supplementary Table 9: SMFA data for Fig S6B and Fig 5G

Feed #	Immunogen	Adjuvant	Sera dilution	Mean oocyst	TRA (%)
#192-2021	*FCS control*			*8.6*	
	6C.WT I53-50::2obx	AddaVax	9	5.2	39.8
	6C.sAgE7 I53-50::2obx	AddaVax	9	8.0	7.0
	6C.mAgE2 I53-50::2obx	AddaVax	9	3.8	55.6
	6C.WT I53-50	AddaVax	9	4.2	51.5
	6C.WT I53-50	AddaVax	27	9.6	-12.3
	6C.sAgE7 I53-50	AddaVax	9	0	100
	6C.sAgE7 I53-50	AddaVax	27	0	100
	6C.mAgE2 I53-50	AddaVax	9	0	100
	6C.mAgE2 I53-50	AddaVax	27	0	100
	6C.mAgE2 HpFerritin	AddaVax	9	0	100
	6C.mAgE2 HpFerritin	AddaVax	27	0	100
#013-2022	*FCS control*			*16.4*	
	6C.WT I53-50::2obx	AddaVax	9	13.7	16.8
	6C.sAgE7 I53-50::2obx	AddaVax	9	20.0	-21.6
	6C.mAgE2 I53-50::2obx	AddaVax	9	9.8	40.2
	6C.WT I53-50	AddaVax	9	13.6	17.4
	6C.sAgE7 I53-50	AddaVax	9	0	100
	6C.sAgE7 I53-50	AddaVax	27	0.1	99.7
	6C.sAgE7 I53-50	AddaVax	81	0.8	95.4
	6C.sAgE7 I53-50	AddaVax	243	8.0	51.5
	6C.mAgE2 I53-50	AddaVax	9	0	100
	6C.mAgE2 I53-50	AddaVax	27	0	100
	6C.mAgE2 I53-50	AddaVax	81	0	100
	6C.mAgE2 I53-50	AddaVax	243	2.0	87.8
	6C.mAgE2 HpFerritin	AddaVax	9	0	100
	6C.mAgE2 HpFerritin	AddaVax	27	0	100
	6C.mAgE2 HpFerritin	AddaVax	81	0.8	95.4
	6C.mAgE2 HpFerritin	AddaVax	243	6.5	60.7

#024-2022	*FCS control*			*14.1*	
	6C.WT I53-50	AddaVax	9	15.6	-10.5
	6C.WT I53-50	AddaVax	27	18.2	-29.0
	6C.sAgE7 I53-50	AddaVax	81	3.4	76.2
	6C.sAgE7 I53-50	AddaVax	243	13.9	1.6
	6C.mAgE2 I53-50	AddaVax	81	1	92.9
	6C.mAgE2 I53-50	AddaVax	243	7.3	48.1
	6C.mAgE2 HpFerritin	AddaVax	81	1.5	86.7
	6C.mAgE2 HpFerritin	AddaVax	243	13.7	3.0

Supplementary Table 10: Calculated TRA estimates for Fig S6B and Fig 5G

Immunogen	Adjuvant	Sera dilution	Calculated % TRA (95% confidence intervals)	p-value
6C.WT I53-50::2obx	AddaVax	9	28.1 (5.7-45.1)	0.009
6C.sAgE7 I53-50::2obx	AddaVax	9	-7.2 (-41.5-18.8)	0.688
6C.mAgE2 I53-50::2obx	AddaVax	9	47.7 (30.2-60.7)	0.000
6C.WT I53-50	AddaVax	9	20.0 (1.0-35.4)	0.020
6C.WT I53-50	AddaVax	27	-19.5 (-53.7-7.0)	0.918
6C.sAgE7 I53-50	AddaVax	9	100 (100-100)	0.000
6C.sAgE7 I53-50	AddaVax	27	99.8 (98.6-100)	0.000
6C.sAgE7 I53-50	AddaVax	81	86.7 (81.0-90.6)	0.000
6C.sAgE7 I53-50	AddaVax	243	27.6 (5.2-44.7)	0.010
6C.mAgE2 I53-50	AddaVax	9	100 (100-100)	0.000
6C.mAgE2 I53-50	AddaVax	27	100 (100-100)	0.000
6C.mAgE2 I53-50	AddaVax	81	96.7 (94.5-98.0)	0.000
6C.mAgE2 I53-50	AddaVax	243	70.3 (59.8-78.0)	0.000
6C.mAgE2 HpFerritin	AddaVax	9	100 (100-100)	0.000
6C.mAgE2 HpFerritin	AddaVax	27	100 (100-100)	0.000
6C.mAgE2 HpFerritin	AddaVax	81	92.3 (88.5-94.9)	0.000
6C.mAgE2 HpFerritin	AddaVax	243	34.1 (14.5-49.3)	0.001
