# Supplementary material for: Vaccination with a structure-based stabilized version of malarial antigen Pfs48/45 elicits ultra-potent transmission-blocking antibody responses
Source: Immunity. 2022 Sep 13;55(9):1680–1692.e8. doi: 10.1016/j.immuni.2022.07.015 (PMC9487866; doi:10.1016/j.immuni.2022.07.015)
Supplement: Document S2. Article plus supplemental information [file mmc2.pdf]

Immunity

Vaccination with a structure-based stabilized version of malarial antigen Pfs48/45 elicits ultra-potent transmission-blocking antibody responses

Graphical abstract

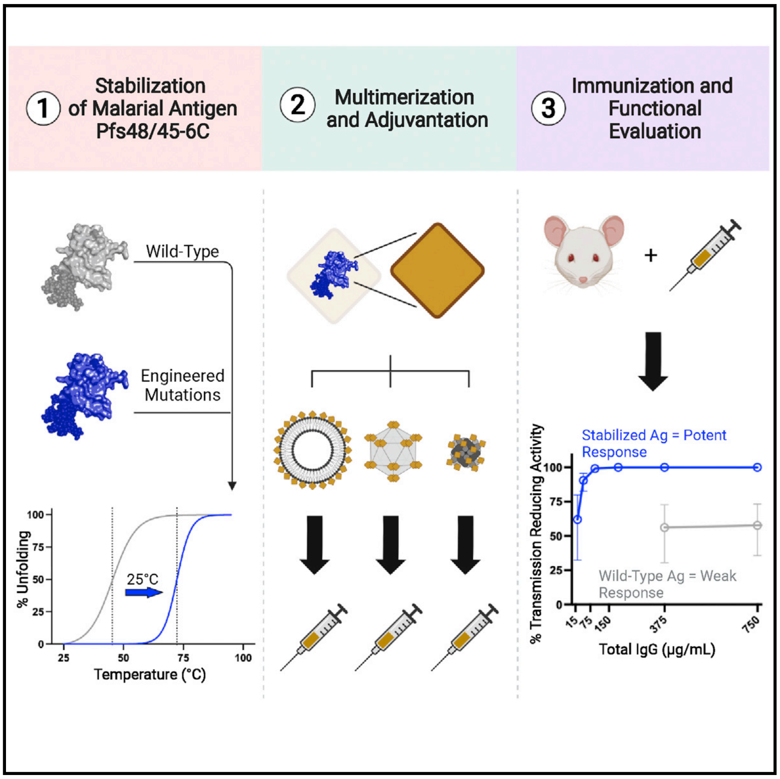

Authors

Brandon McLeod,
Moustafa T. Mabrouk,
Kazutoyo Miura, ..., Neil P. King,
Jonathan F. Lovell,
Jean-Philippe Julien

Correspondence

jean-philippe.julien@sickkids.ca

In brief

Clinical development of transmission-blocking malaria vaccine Pfs48/45 antigens has been hindered by its poor biochemical characteristics. McLeod et al. utilized protein engineering techniques to improve the stability and biophysical properties of Pfs48/45-6C, which enhanced the inhibitory capacity of the immune response by 1–2 orders of magnitude, compared with the wild-type antigen across three vaccine platforms.

Highlights

- Combinatorial structure-based engineering of Pfs48/45-6C antigen improves its stability
- Thermostability and recombinant protein yield are increased over WT antigen
- 2.18 Å resolution crystal structure provides molecular insights into stability gain
- Multimerized stabilized antigens elicit potent transmission-reducing activity *in vivo*

McLeod et al., 2022, Immunity *55*, 1680–1692
September 13, 2022 © 2022 The Author(s). Published by Elsevier Inc.
https://doi.org/10.1016/j.immuni.2022.07.015

Article

Vaccination with a structure-based stabilized version of malarial antigen Pfs48/45 elicits ultra-potent transmission-blocking antibody responses

Brandon McLeod,[1,2] Moustafa T. Mabrouk,[3] Kazutoyo Miura,[4] Rashmi Ravichandran,[5,6] Sally Kephart,[7] Sophia Hailemariam,[1,2] Thao P. Pham,[4] Anthony Semesi,[1] Iga Kucharska,[1] Prasun Kundu,[1] Wei-Chiao Huang,[3] Max Johnson,[5,6] Alyssa Blackstone,[5,6] Deleah Pettie,[5,6] Michael Murphy,[5,6] John C. Kraft,[5,6] Elizabeth M. Leaf,[5,6] Yang Jiao,[3] Marga van de Vegte-Bolmer,[8] Geert-Jan van Gemert,[8] Jordache Ramjith,[9] C. Richter King,[10] Randall S. MacGill,[10] Yimin Wu,[10] Kelly K. Lee,[7] Matthijs M. Jore,[8] Neil P. King,[5,6] Jonathan F. Lovell,[3] and Jean-Philippe Julien[1,2,11,12,*]

[1]Program in Molecular Medicine, The Hospital for Sick Children Research Institute, 686 Bay Street, Toronto, ON M5G 0A4, Canada
[2]Department of Biochemistry, University of Toronto, 1 King's College Circle, Toronto, ON M5S 1A8, Canada
[3]Department of Biomedical Engineering, University at Buffalo, State University of New York, Buffalo, NY 14260, USA
[4]Laboratory of Malaria and Vector Research, National Institute of Allergy and Infectious Diseases, National Institutes of Health, 12735 Twinbrook Parkway, Rockville, MD 20852, USA
[5]Department of Biochemistry, University of Washington, Seattle, WA 98195, USA
[6]Institute for Protein Design, University of Washington, Seattle, WA 98195, USA
[7]Department of Medicinal Chemistry, University of Washington, Seattle, WA 98195, USA
[8]Department of Medical Microbiology, Radboud University Medical Center, Nijmegen, the Netherlands
[9]Radboud Institute for Health Sciences, Department for Health Evidence, Biostatistics Section, Radboud University Medical Center, Nijmegen, the Netherlands
[10]PATH's Malaria Vaccine Initiative, 455 Massachusetts Avenue NW Suite 1000, Washington, DC 20001, USA
[11]Department of Immunology, University of Toronto, 1 King's College Circle, Toronto, ON M5S 1A8, Canada
[12]Lead contact
*Correspondence: jean-philippe.julien@sickkids.ca
https://doi.org/10.1016/j.immuni.2022.07.015

SUMMARY

Malaria transmission-blocking vaccines (TBVs) aim to elicit human antibodies that inhibit sporogonic development of *Plasmodium falciparum* in mosquitoes, thereby preventing onward transmission. Pfs48/45 is a leading clinical TBV candidate antigen and is recognized by the most potent transmission-blocking monoclonal antibody (mAb) yet described; still, clinical development of Pfs48/45 antigens has been hindered, largely by its poor biochemical characteristics. Here, we used structure-based computational approaches to design Pfs48/45 antigens stabilized in the conformation recognized by the most potently inhibitory mAb, achieving >25°C higher thermostability compared with the wild-type protein. Antibodies elicited in mice immunized with these engineered antigens displayed on liposome-based or protein nanoparticle-based vaccine platforms exhibited 1–2 orders of magnitude superior transmission-reducing activity, compared with immunogens bearing the wild-type antigen, driven by improved antibody quality. Our data provide the founding principles for using molecular stabilization solely from antibody structure-function information to drive improved immune responses against a parasitic vaccine target.

INTRODUCTION

Malaria is a major global health burden, with half the world population at risk of infection and 241 million confirmed cases in 2020 alone, resulting in more than 627,000 annual deaths predominantly in children (World Health Organization, 2021). Notwithstanding that substantial progress has been made in recent decades to reduce malaria incidence, this progress has largely slowed down in recent years, hastening the need for additional and innovative interventions (Talapko et al., 2019; World Health Organization, 2021). Malaria is caused by infection from vector-borne parasites of the genus *Plasmodium*, with the overwhelming majority of the clinical burden being driven by *Plasmodium falciparum* (Pf) (World Health Organization, 2021). A broadly effective malaria vaccine against Pf has remained elusive with only a single vaccine, RTS,S/AS01, having recently been approved for use after pilot implementation studies in Malawi, Ghana, and Kenya (The RTS S Clinical Trials Partnership, 2012; Duffy and Patrick Gorres, 2020; PAHO/WHO|Pan American Health Organization, 2021). RTS,S/AS01 is an anti-infection vaccine that aims to protect from parasite exposure. However, RTS,S/AS01 vaccine efficacy is modest,

 © 2022 The Author(s). Published by Elsevier Inc.
This is an open access article under the CC BY license (http://creativecommons.org/licenses/by/4.0/).

and protection from severe disease falls below 35% after 1 year across age groups (White et al., 2015), highlighting a need for improved and complementary biomedical countermeasures against malaria.

The complexity of the Pf life cycle, featuring various stages that each present their own proteomic profile (Florens et al., 2002), is a challenge for the development of highly efficacious vaccines; however, it also presents a unique opportunity to target multiple essential proteins across life stages to curb the infection-transmission cycles within communities. Indeed, a previous study demonstrated the potential for synergistic inhibition of the Pf infection-transmission cycle when combining an anti-infective antibody with an antibody targeting the sexual stage of the parasite—the two mosquito vector-host bottlenecks of transmission (Sherrard-Smith et al., 2018). By targeting essential sexual stage antigens or mosquito antigens, transmission-blocking vaccines (TBVs) seek to induce antibodies in humans that are capable of blocking Pf transmission to *Anopheles* mosquito vectors (Miura et al., 2013; Delves et al., 2018; Acquah et al., 2019). In pre-clinical and clinical studies, TBVs have elicited strong antibody responses capable of greater than 90% transmission-reducing activity (TRA) in some humans (Healy et al., 2021) (NCT02334462, NCT03917654). Hence, TBVs have the potential to be used as an adjunct to anti-infective vaccines to help reduce malaria transmission and drive global elimination efforts (Brod et al., 2018).

The quest toward finding the best TBV antigen for vaccine development has in part been driven by monoclonal antibody (mAb) research (Julien and Wardemann, 2019). The most potent Pf transmission-blocking mAb yet identified is against Pfs48/45, with an IC_{80} (antibody concentration that gives 80% TRA) of ~1 μg/mL (Kundu et al., 2018). mAb TB31F is a humanized mAb with its parent elicited by whole gametocyte immunization in rats, and it is currently undergoing evaluation in a phase 1 clinical trial (NCT04238689) to determine its safety and Pf transmission-reducing potential in humans, with encouraging interim data (van der Boor et al., 2020). By contrast, the most potent mAbs reported to date against other clinical-stage TBVs have overall been substantially less potent (Pfs25: mAb 2544 has an IC_{80} of ~16 μg/mL [McLeod et al., 2019]; Pfs230: mAb LMIV230-01 required ~60 μg/mL to achieve ~80% TRA [Coelho et al., 2021]). Furthermore, it has been demonstrated that polyclonal antibody responses elicited in natural infection against Pfs48/45 can mediate transmission inhibition (Stone et al., 2016, 2018).

Pfs48/45 is a relatively conserved glycophosphatidylinositol (GPI)-linked protein expressed on the surface of gametes and is assumed to be essential for male gametocyte fertility (van Dijk et al., 2001). Developing a Pfs48/45-based TBV has proven challenging, largely due to the difficulties of recombinantly expressing Pfs48/45 in sufficient quality and quantity (Outchkourov et al., 2008). The most advanced Pfs48/45 antigen is genetically fused to the R0 protein for enhanced expression, folding, and stabilization of the Pfs48/45-6C domain (Outchkourov et al., 2008; Acquah et al., 2017; Singh et al., 2021). Although this antigen recently entered a phase 1 clinical trial (NCT04862416), it remains unclear whether stabilization of Pfs48/45 through fusion to another protein can be sufficient to elicit the most potent transmission-blocking activity against this target.

In this study, we evaluated whether structure-guided immunogen design—a computational approach that has previously been employed primarily to deliver improved antibody responses elicited by immunogens based on the fusion machinery of viral pathogens (McLellan et al., 2013; Pallesen et al., 2017)—could be utilized to stabilize the Pfs48/45-6C domain in its conformation recognized by the most potent transmission-blocking mAb, TB31F, and lead to improved TRA against malaria Pf. We report the design of stabilized Pfs48/45 antigens with improved expression yield and biochemical properties that elicit exquisitely potent malaria transmission-blocking activity. These findings highlight a transformative role for structure-based immunogen design and provide the founding principles of its use for parasitic vaccine targets.

RESULTS

Structure-based engineering improves Pfs48/45-6C stability

Structure-guided protein engineering efforts were undertaken to increase the thermostability of domain III, or the 6C domain, of Pfs48/45 based on its structure in complex with the most potent transmission-blocking mAb yet described, TB31F (Kundu et al., 2018; Figures 1A and 1B). Visual inspection (i.e., manually scanning through regions of the protein and assessing the molecular interactions of residues in their environment) of the crystal structure in Molecular Operating Environment (MOE) (Chemical Computing Group, 2022) identified 12 candidate residues for mutation outside the TB31F epitope that may stabilize the 6C fold. When recombinantly expressed and tested, only one mutation, G397L, notably increased the melting temperature (T_m) of the antigen by nearly 10°; from 46.9°C ± 2.5°C to 55.5°C ± 1.3°C (Figure 1C). Encouraged by this finding, a model of 6C.G397L was used in conjunction with Rosetta (Das, and Baker, 2008) to further improve its thermostability. Among approximately 4,000 point mutations generated *in silico*, the majority of top-ranking stability mutations were concentrated in four distinct regions outside the TB31F epitope (Figure 1B). From this panel of *in silico*-identified mutations, seven 6C-single-antigen-engineered ("6C.sAgE") constructs were generated and recombinantly expressed (Table 1). The variants exhibited a range of altered melting temperatures, with 6C.sAgE7 (G397L + H308Y) being the most improved with a T_m of 69.8°C ± 0.3°C (Figures 1C and 1D; Table 1). In addition to its marked improvement in melting temperature, 6C.sAgE7 also exhibited a reduced tendency to aggregate, eluting from size-exclusion chromatography (SEC) in a predominantly monodisperse peak in contrast to wild-type 6C (6C.WT), which contained a large quantity of higher-order misfolded oligomers (Figure 1E). In an accelerated thermostability study of 4 weeks (28 days) across three different temperatures (−20°C, 4°C, and 40°C) (Figure 1F), 6C.sAgE7 exhibited (1) no detectable loss of secondary structure, (2) no aggregation or degradation, and (3) no loss of binding to mAb TB31F, indicating that 6C.sAgE7 has an outstanding ability to maintain its stabilized structure even under prolonged thermal stress (Figures 1G, 1H, and S1). In brief, the engineered modifications made to 6C.sAgE7 substantially improved its overall biophysical characteristics.

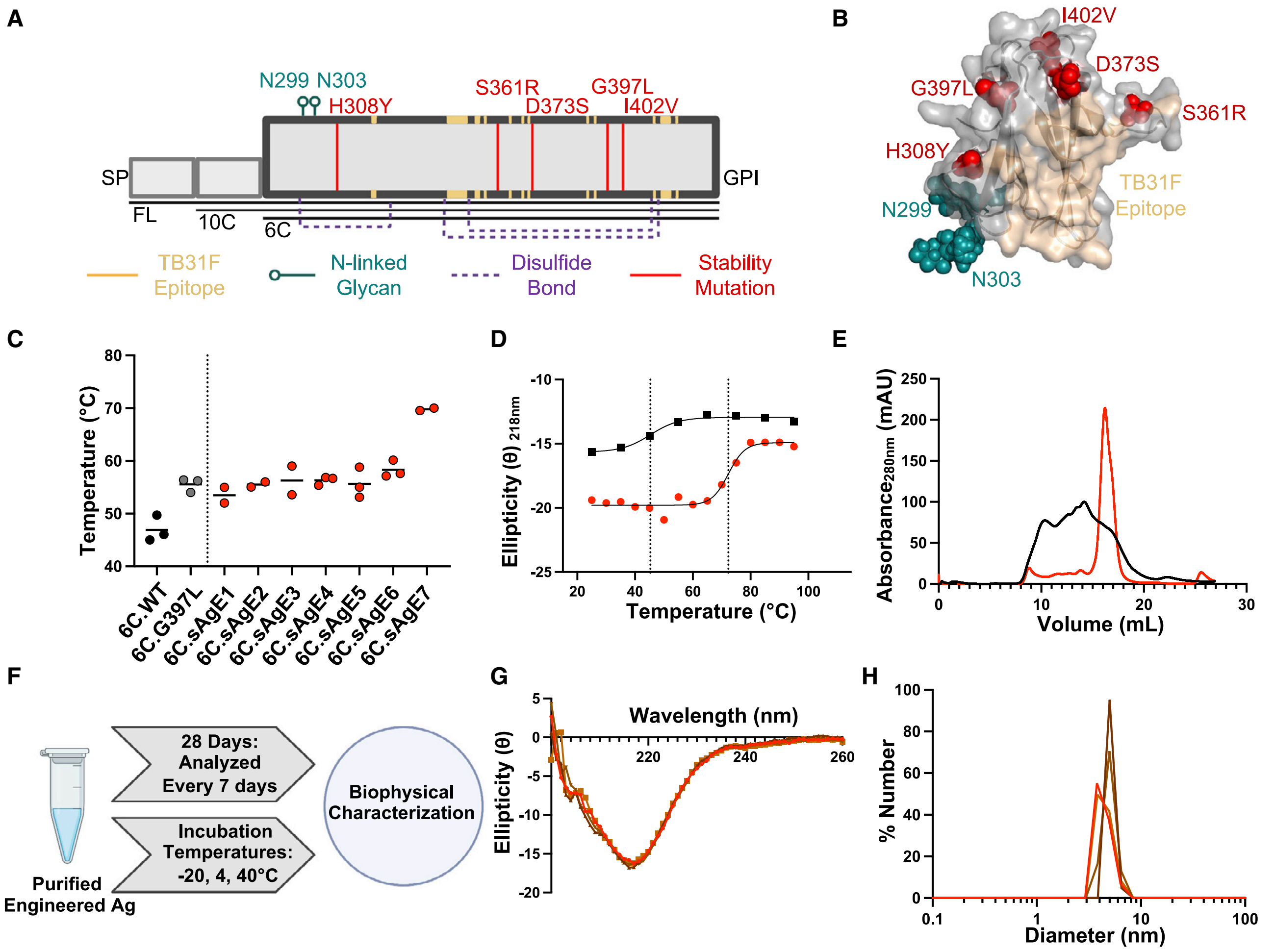

Figure 1. Structure-based design stabilizes Pfs48/45-6C antigens

(A) Schematic of Pfs48/45-6C region (not to scale). Residues of the TB31F epitope are in beige at the border of the 6C domain; residues with associated N-linked glycans are in teal; disulfide bonds are in purple; and engineered stability mutations are in red. SP, signal peptide; GPI, glycophosphatidylinositol anchor; FL, full-length; 10C, two domains of Pfs48/45 that contain 10 cysteines; 6C, six cysteine single domain.

(B) Three-dimensional model highlighting the information in (A).

(C) Thermostability as measured by CD during temperature ramp. For each protein, assays were performed in duplicates or triplicates (dots) and the mean (bar) is shown.

(D) Representative CD melting curve observed at 218 nm. 6C.WT is denoted by black squares and 6C.sAgE7 by red circles. Dotted lines mark the inflection point in the melting curve, corresponding to T_m.

(E) Superimposed representative SEC chromatographs of 6C.WT (black) and 6C.sAgE7 (red).

(F) Overview of accelerated thermostability study.

(G) CD spectra of 6C.sAgE7 on day 0 (red), and week 4, at -20°C (dark brown), 4°C (medium brown), and 40°C (light brown).

(H) DLS spectra of 6C.sAgE7 on day 0 (red), and week 4, at -20°C (dark brown), 4°C (medium brown), and 40°C (light brown).

See also Figure S1.

The most stabilized Pfs48/45-6C antigen elicits antibodies that drive potent TRA

Next, we evaluated how these improved biophysical properties translated into immunogenicity and Pf TRA. Groups of six CD-1 mice were immunized with a 5 μg dose of differentially stabilized Pfs48/45-6C antigens (6C.WT, 6C.sAgE3, 6C.sAgE5, and 6C.sAgE7) adjuvanted with ISA720 and boosted at 21 days before sera were harvested on day 42 for evaluation (Figure 2A). In addition, the same Pfs48/45-6C antigens were formulated on the CoPoP multimerization platform (Huang et al., 2018) by binding His-tagged antigens to cobalt-containing "CPQ" immunogenic liposomes (Figure S2A), which also co-incorporate synthetic monophosphoryl lipid A and the saponin QS-21 as adjuvants. Antigen binding was associated with conserved overall CoPoP particle size as assessed by dynamic light scattering, with particle size remaining below 150 nm for all samples, and with a polydispersity index of less than 0.2, reflecting a relatively monodisperse population (Figure S2B). As determined by fluorescence energy transfer, labeled 6C.WT antigen rapidly bound to CoPoP

CellPress
OPEN ACCESS

Table 1. Rosetta modeling statistics and associated biophysical parameters for Pfs48/45-6C variants

Pfs48/45-6C variant	Mutations	Rosetta score	T_m (°C)	TB31F binding K_D (nM)
6C.WT	N/A	N/A	46.9 ± 2.5	3.1 ± 2.3
6C.G397L	G397L	N/A	55.5 ± 1.3	5.4 ± 2.5
6C.sAgE1	G397L, E365K	-66.72	53.5 ± 2.1	2.5 ± 1.8
6C.sAgE2	G397L, S361R	-65.04	55.5 ± 0.7	3.3 ± 1.8
6C.sAgE3	G397L, I402V	-63.32	56.3 ± 3.8	1.6 ± 0.6
6C.sAgE4	G397L, D373S	-62.69	56.3 ± 0.8	1.8 ± 0.1
6C.sAgE6	G397L, I398V	-72.15	58.3 ± 1.6	2.3 ± 0.2
6C.sAgE5	G397L, I378V	-71.38	55.7 ± 2.9	2.1 ± 0.8
6C.sAgE7	G397L, H308Y	-69.20	69.8 ± 0.3	1.1 ± 0.6
6C.mAgE1	G397L, H308Y, I402V	N/A	72.2 ± 0.3	2.1 ± 0.1
6C.mAgE2	G397L, H308Y, I402V, S361R, D373S	N/A	72.3 ± 0.7	2.0 ± 0.4

liposomes but not liposomes lacking cobalt within the porphyrin-phospholipid, and the liposome-displayed antigens maintained complexation stability over time during incubation with serum (Figures S2C and S2D).

Among adjuvant groups, no significant differences in ELISA units (EUs) were observed ($p > 0.137$) between the various Pfs48/45-6C antigens, despite their differences in thermostability (Figure 2B). For the same antigen, EUs were significantly higher ($p < 0.011$) in the CPQ formulated groups compared with the ISA720 adjuvanted groups, except for 6C.sAgE3, for which only a higher trend was observed ($p = 0.095$). Overall, this data indicate that the formulation of Pfs48/45-6C constructs with CoPoP liposomes contributed to increased antibody titers (Figure 2B). Next, standard membrane feeding assay (SMFA) experiments were performed on NF54 isolates to assess parasite TRA. Whereas purified IgG from the 6C.WT, 6C.sAgE3, and 6C.sAgE5 groups tested at 750 μg/mL showed moderate parasite inhibition capacity in the 30%–80% TRA range, purified IgG from the 6C.sAgE7 group showed complete blocking, 100% TRA, at this concentration (Figure 2C). Thus, this data suggest that the higher thermostability for the 6C.sAgE7 antigen leads to improved functional activity by the elicited antibody response (Figures 2D and S3; Table 1). Anti-6C.sAgE7 IgG was evaluated at multiple concentrations in SMFA, and IC_{80} value in the ISA720 adjuvanted group was 2,594 EU, whereas the IC_{80} value was almost half, 1,098 EU, in the CPQ adjuvant group (Figure 2E). Taken together, this data suggest that both the conformational stability of the antigen and its multimeric formulation in CPQ contributed to the high-quality antibody response (i.e., antibody showed higher functional activity measured by SMFA at the same mass concentration of total or antigen-specific IgG).

Structural characterization reveals molecular basis of enhanced stability

Next, we investigated whether we could combine stability mutations from our top *in silico* and experimentally validated hits to create a Pfs48/45-6C antigen with further improved biophysical characteristics over 6C.sAgE7. The 6C multiple antigen engineered 1 construct, or 6C.mAgE1, combined the 6C.sAgE7 mutations (G397L + H308Y) together with another highly ranked stabilizing mutation, I402V, whereas 6C.mAgE2 contained these three and two additional mutations based on their favorable biophysical characteristics as individual mutants and compatible co-localization in the antigen: S361R + D373S (Figures 3A–3C; Table 1). Both 6C.mAgE1 and 6C.mAgE2 had marginally improved thermostability from 6C.sAgE7, with T_ms of 72.2°C and 72.3°C, respectively (Figure 3D). However, recombinant expression of 6C.mAgE1 and 6C.mAgE2 by transient transfection in HEK293F cells resulted in drastic improvements in the yields of well-folded, secreted antigen in the supernatant, compared with less engineered antigens (Figure 3E); and >30-fold improvements in final yields after purification, compared with WT antigen; and >4-fold when compared with 6C.sAgE7 (Figure 3F).

The X-ray crystal structure of 6C.mAgE1 at 2.18 Å resolution was obtained and provides molecular insights into the combinatorial stabilizing potential of its mutations in Pfs48/45-6C (Table S1). The H308Y mutation allows increased van der Waals interactions with the nearby I393 residue and additionally puts a hydroxyl in H-bonding distance to D382, likely contributing to greater overall fold stability in this region (Figure 3B, inset I). The G397L mutation introduces a branched hydrocarbon side chain in a previously empty hydrophobic pocket, allowing for improved van der Waals contacts with neighboring residues N337, L339, E382, and V399 (Figure 3B, inset II). I402V reduces by one methyl the hydrocarbon content of the side chain at this position, alleviating sterics in an otherwise densely packed hydrophobic pocket made by the side chains of residues Y336, I376, I378, I398, and I425 (Figure 3B, inset III).

To assess whether there were differences in the structural dynamics between 6C.sAgE7 and 6C.mAgE2, and thus an impact of combining selected engineered mutations, these proteins were analyzed by hydrogen-deuterium exchange mass spectrometry (HDX-MS; Table S2). Because of its low yield and poor stability, 6C.WT could not be obtained in sufficient amount and purity to analyze by HDX-MS as a comparator. The two stabilized antigens were highly similar, with only minor differences largely within the experimental margin of error for this technique (Figure 3G). In an accelerated thermostability stress study, both 6C.mAgE1 and 6C.mAgE2 showed no signs of aggregation or degradation, further confirming their exquisite stability (Figure S1). Combined, these structural insights provide a molecular understanding for the improved biophysical and functional improvements of the engineered Pfs48/45-6C antigens.

High TRA is associated with an extended Pfs48/45 antigenic surface

With the improved biophysical characteristics associated with 6C.mAgE2, we next sought to evaluate whether presentation of the stabilized TB31F epitope alone was sufficient to achieve high-potency antibodies in immunization, or whether additional regions on Pfs48/45-6C stabilized in this particular antigenic conformation contributed to the potent inhibitory response. For this purpose, we introduced in 6C.mAgE2 four and six

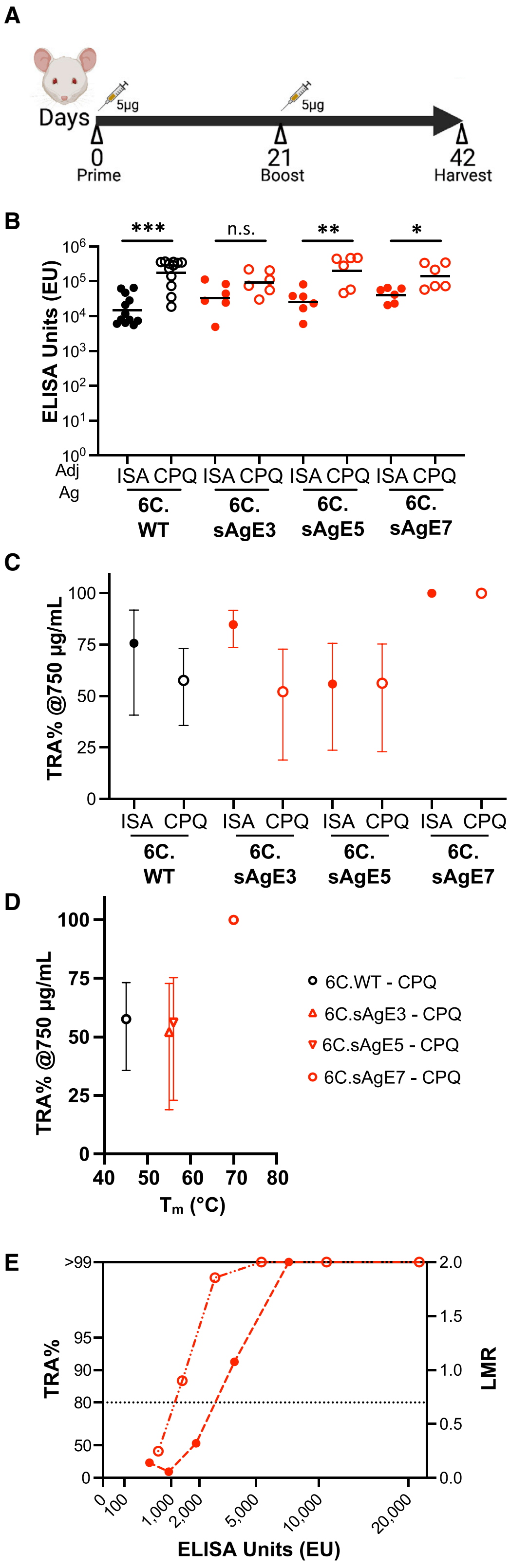

Figure 2. Transmission-blocking antibody responses are elicited by stabilized Pfs48/45-6C variants

(A) Mouse immunization schedule.

(B) Serum anti-6C.sAgE7 ELISA units (EUs) of ISA720 adjuvanted (solid) and CPQ-formulated (hollow) Pfs48/45-6C variants in red, and 6C.WT in black. Each dot corresponds to individual mice, the bar represents geometric mean. 6C.WT proteins were tested in two independent immunization studies, while the others were in a single study. n.s. not significant ($p > 0.05$); *, **, and *** indicate $p \leq 0.05$, $p \leq 0.01$, and $p \leq 0.001$, respectively, as determined by unpaired Student's t test for log-transformed EU.

(C) SMFA of purified IgG at 750 µg/mL. The symbols represent the best-estimate percent inhibition in oocyst density (TRA%) from two independent assays, with error plotted as 95% confidence interval (CI), except for IgG from 6C.WT with ISA720 group, which was tested in one assay, and the IgG from 6C.WT with CPQ, which was tested in three assays.

(D) SMFA with purified IgG from CPQ-formulated antigens in (C), plotted as a function of thermostability (T_m). 6C.sAgE3 and 6C.sAgE5 have identical T_m values of 56°C but are offset for graphical presentation.

(E) SMFA as a function of 6C.sAgE7 specific ELISA units for purified IgG from both ISA720 adjuvanted (solid red) and CPQ-formulated (hollow red) 6C.sAgE7-immunized sera. SMFA data from each feed are shown in Tables S3 and S4.

See also Figure S2.

non-native N-linked glycosylation sites (6C.mAgE2+4Gly and 6C.mAgE2+6Gly) to mask the majority of the surface of 6C.mAgE2 without interfering with the TB31F epitope (Figure 4A). As intended, addition of these immune-masking N-linked glycans did not impede folding of the antigens, which retained T_ms greater than 60°C and high-affinity binding to TB31F (Figures S4C and S4D).

As above, mice were immunized twice with 5 µg of 6C.mAgE2, 6C.mAgE2+4Gly, or 6C.mAgE2+6Gly displayed on the CPQ liposome platform (Figure 2A). All groups elicited similarly high EUs against 6C.sAgE7 ($p = 0.646$, Figure 4B). ELISA against 6C.mAgE2+6Gly and a TB31F epitope-knockout construct (6C.TB-KO) showed that sera elicited by 6C.mAgE2+4Gly and 6C.mAgE2+6Gly were proportionally significantly more reactive ($p < 0.05$) to 6C.mAgE2+6Gly than 6C.TB-KO, compared with sera from 6C.WT-, 6C.sAgE7-, or 6C.mAgE2-immunized animals (Figure S5), indicating 6C.mAgE2+4Gly and 6C.mAgE2+6Gly antisera recognized more the canonical TB31F epitope or newly introduced immune-masking-associated glycan epitopes. While the purified IgG from the 6C.mAgE2 group showed 100% TRA in SMFA at 375 µg/mL, purified IgG from the 6C.mAgE2+4Gly and 6C.mAgE2+6Gly groups dropped sharply to 86.6% and 38.3%, respectively (Figure 4C), indicating that the introduction of masking N-linked glycans across the antigenic surface of Pfs48/45-6C decreased the potency of the polyclonal response. These data imply that the TB31F epitope alone is not sufficient for potent transmission reduction.

To better understand the potency of the Pfs48/45 antigenic surface that surrounds the TB31F epitope, we next used a recently characterized mAb derived from natural infection, RUPA-117, which has an IC_{80} of approximately 10 µg/mL and binds to a nearby but distinct epitope from TB31F (Fabra-García et al., 2021). Consistent with modeling suggesting that the additional glycans introduced in the hyperglycosylated constructs would impede binding to this recently discovered potent epitope, mAb RUPA-117 showed markedly reduced binding to 6C.mAgE2+4gly, and this binding was further perturbed for

CellPress
OPEN ACCESS

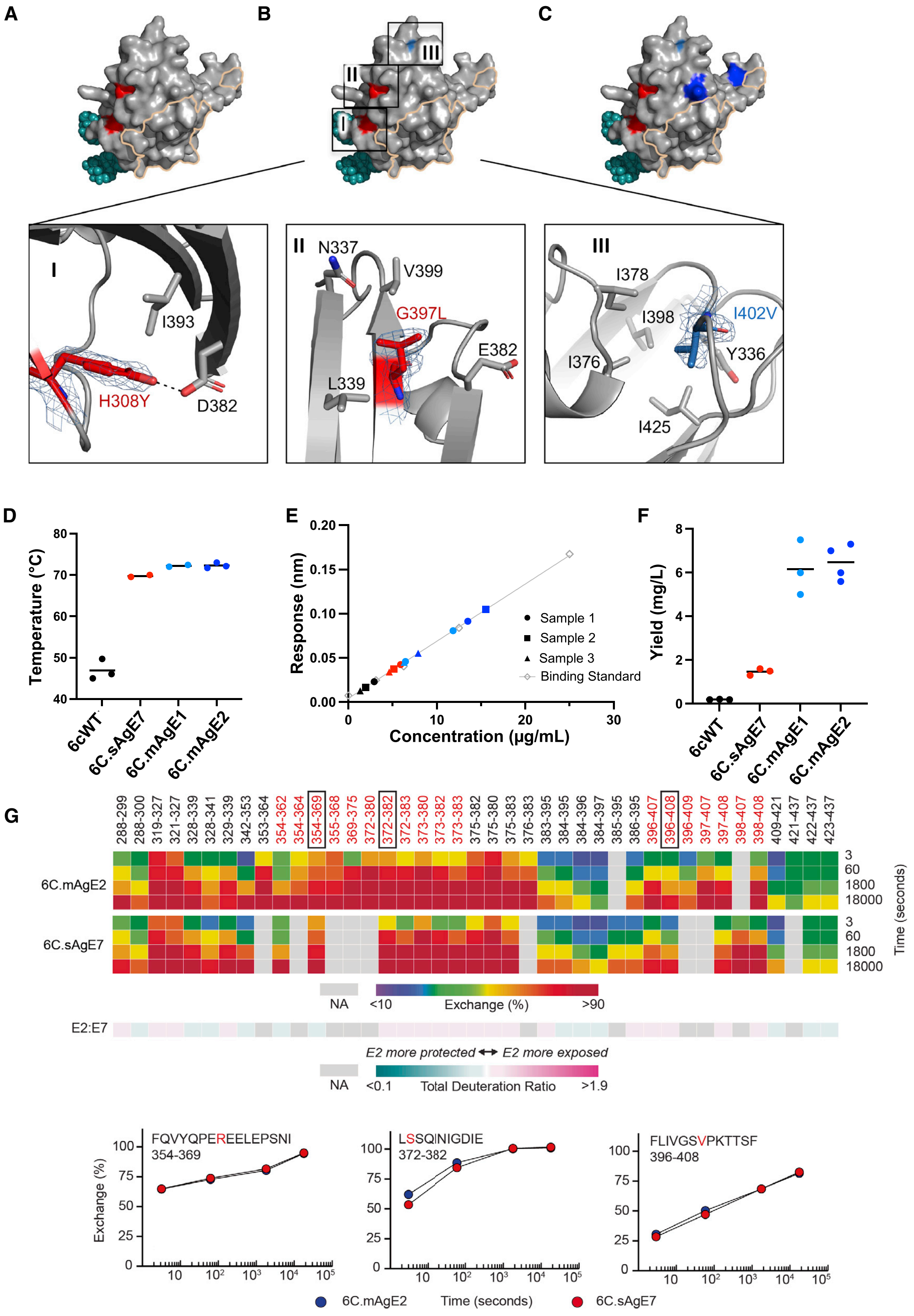

Figure 3. Molecular insights provide basis for enhanced biochemical properties

(A–C) Surface representation of (A) 6C.sAgE7, (B) 6C.mAgE1, and (C) 6C.mAgE2 with modeled N-linked glycans in teal, 6C.sAgE7 thermostability mutations in red, the I402V mutation in pale blue, and the S361R and D373S mutations in dark blue. The TB31F epitope is outlined in beige. Boxes zoom in on associated

(legend continued on next page)

6C.mAgE2+6Gly (Figure 4D). Taken together, our data demonstrate that an expanded antigenic surface on Pfs48/45 beyond the canonical TB31F epitope, but stabilized in the conformation recognized by TB31F, is best to elicit polyclonal sera with the strongest possible TRA.

Engineered Pfs48/45-6C antigens elicit potent transmission-reducing antibodies across multimerization platforms

Next, we sought to determine the extent to which the stabilized Pfs48/45-6C antigens could improve the potency of the elicited transmission-reducing antibody response, compared with the WT antigen. Purified IgG from mice immunized with 5 μg of 6C.WT, 6C.sAgE7, 6C.mAgE1, and 6C.mAgE2 displayed on the CPQ liposome platform (Figures 5A and 5B) was tested in SMFA experiments with serial dilutions ranging from 1,500 to 23 μg/mL. While all antibodies elicited by the engineered constructs with CPQ adjuvant showed almost complete inhibitions (>99% TRA) at 375 μg/mL, 6C.WT antibodies had a TRA of only 62.7% (95% CI, 20.4–81.6; p = 0.009) even at 1,500 μg/mL (Figure 5C). The IC_{80}'s were calculated as 39.4 μg/mL (95% CI; 31.0–48.8), 33.7 μg/mL (95% CI; 15.7–58.3), and 57.7 μg/mL (95% CI; 35.1–86.0) for 6C.sAgE7, 6CmAgE1, and 6C.mAgE2, respectively (Figure 5C). These data revealed a stark difference in potency and a gain of at least one to two logs of functional activity for the antibodies elicited by the engineered antigens as compared with those elicited by 6C.WT.

The 6C.WT, 6C.sAgE7, and 6C.mAgE2 antigens were also genetically fused to the trimeric component of the two-component I53-50A protein nanocage (Brouwer et al., 2019), and 6C.mAgE2 to *Helicobacter pylori* (Hp) ferritin protein nanocage (Wuertz et al., 2021; Figure 5D). Mice were immunized with 5 μg immunogen formulated in Addavax at days 0 and 28, before harvesting serum at day 42. An appreciable increase in titers and TRA across antigens was observed when compared with non-assembling (trimeric) I53-50 controls, demonstrating the superiority of the antibody response elicited by multimeric immunogens (Figures S6A and S6B). Similarly to what was observed with the CoPoP platform (Figure 5B), all nanocage groups exhibited high antibody titers following the boost without significant differences between the I53-50 and HpFerritin nanocages (Figure 5F, p = 0.8481). Both the 6C.sAgE7 and 6C.mAgE2 I53-50 particles exhibited significantly higher titers than the 6C.WT antigen on the I53-50 particle (p = 0.007 and p = 0.0025, respectively). Whereas 6C.WT fused to the I53-50 particle displayed a low mean TRA of 35.5% with 9-fold serum dilution, the 6C.sAgE7 and 6C.mAgE2 I53-50 particles and 6C.mAgE2-HpFerritin particles exhibited nearly 100% TRA, even at 81-fold serum dilutions, and retained potencies of 51.5%, 87.8%, and 65.7%, respectively, at 243-fold dilutions (Figure 5G). In comparison to the response elicited by 6C.WT-I53-50 particles, this represents at least a log improvement in TRA, consistent with the findings on the CoPoP multimerization platform. Combined, these data demonstrate potent TRA elicited by engineered Pfs48/45-6C constructs across several vaccine multimerization platforms.

DISCUSSION

Despite being the target of the most potent anti-malarial transmission-blocking mAb yet reported, Pfs48/45 has largely remained a challenging target for vaccine development. Its low recombinant expression and intrinsic instability have been the focus of various rescue strategies, including exploration of alternative expression systems, fusion proteins, and multimerization methods (Outchkourov et al., 2008; Acquah et al., 2017; Singh et al., 2021), all with mixed success. Structure-guided protein engineering has emerged as a viable strategy to stabilize antigens, most notably the fusion proteins of enveloped viruses. Prefusion-stabilizing mutations in the respiratory syncytial virus (RSV) F protein (McLellan et al., 2013) and in the MERS and SARS-CoV-2 spike proteins (Pallesen et al., 2017; Corbett et al., 2020; Hsieh et al., 2020) are salient examples of how stabilizing an antigen in the conformation associated with recognition by the most potent mAbs can generate immunogens eliciting enhanced neutralization responses, with demonstrated success for vaccine development. Here, we sought to investigate whether antigen stabilization as a strategy could extend beyond viral fusion glycoproteins and improve the functional immune response against a promising yet challenging antigen from the protozoan parasite *Plasmodium falciparum* (*P. falciparum*).

The Pfs48/45-stabilizing mutations we identified from *in silico* methods stabilize the Pfs48/45-6C fold recognized by

mutations as seen in the 6C.mAgE1 crystal structure: (I) H308Y; (II) G397L; and (III) I402V. Pfs48/45-6C is represented in a gray ribbon, and neighboring side chains are shown. H-bonds are denoted by a dashed black line, and a composite omit electron density map at 1.0 sigma for the mutated residues is shown in blue mesh.

(D) Thermostability of Pfs48/45-6C stabilized variants as determined by CD. Values are plotted from at least two replicates from individual purifications, with the bar as mean.

(E) Quantitation of secreted, well-folded Pfs48/45-6C in supernatant using TB31F Fab-coated biosensors in BLI. The standard curve was generated with known concentrations of purified 6C.mAgE2 serially diluted in supernatant from untransfected HEK293F cells (gray). Antigen quantitation from three independent transient transfections are plotted as data points represented by circles, squares, and triangles, respectively, and are plotted on the interpolated standard curve. Data corresponding to different antigens are colored as in (D).

(F) Recombinant protein yields recovered post-affinity and size exclusion chromatography purification from at least three separate transfections in HEK293F cells, with the bar representing the mean yield.

(G) Percent hydrogen/deuterium exchange is plotted for 6C.mAgE2 and 6C.sAgE7 peptides at multiple time points up to 5 h. Peptides that contain amino acid substitutions are colored in red. The ratio of total deuteration comparing 6C.mAgE2 and 6C.sAgE7 is plotted across the sequence. Peptides shaded teal indicate regions where 6C.mAgE2 is more protected, and peptides shaded pink indicate regions where 6C.mAgE2 is more exposed. Uptake plots are shown for three peptides to highlight comparisons where substitutions were made. Each time point is an average from duplicate measurements, and errors bars of the standard deviation between replicates are plotted, but too small for visualization.

See also Figures S3 and S4 and Tables S1 and S2.

CellPress
OPEN ACCESS

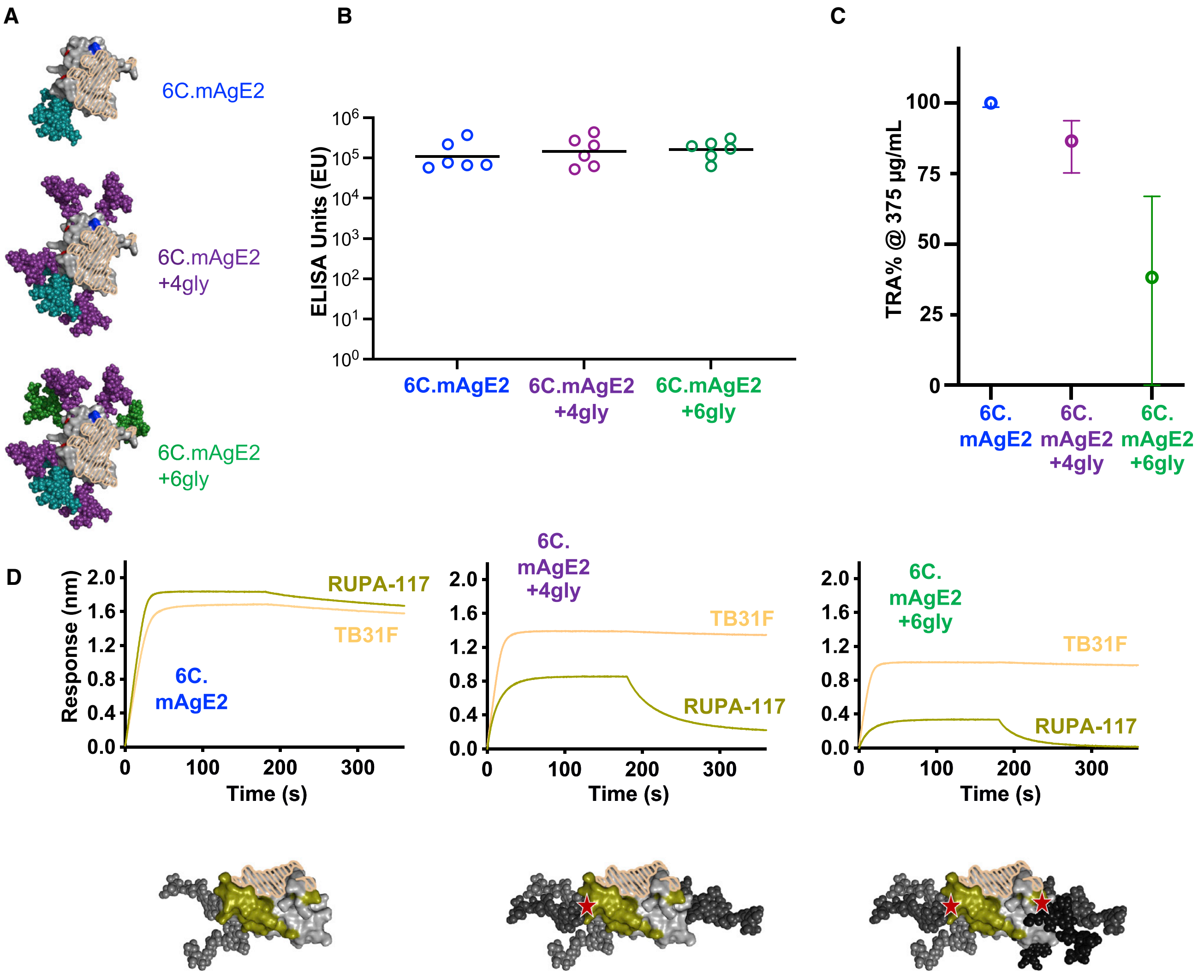

Figure 4. An extended Pfs48/45-6C antigenic surface is associated with potent TRA

(A) Schematics of 6C.mAgE2 hyperglycosylated variants through introduction of non-natural N-linked glycans. Top, 6C.mAgE2, with two N-linked glycans encoded by the native sequence in teal. Mid, 6C.mAgE2+4Gly, with four non-native N-linked glycans introduced in purple. Bottom, 6C.mAgE2+6Gly, with two additional N-linked glycans in green. The TB31F epitope is hatched beige.

(B) Immunogenicity of hyperglycosylated Pfs48/45 variants formulated in CPQ as measured by ELISA against 6C.sAgE7-coated antigen. Values are plotted as individual mice, with horizontal black bar representing the geometric mean.

(C) SMFA of purified IgG elicited by 6C.mAgE2, 6C.mAgE2+4Gly, and 6C.mAgE2+6Gly, at 375 µg/mL. Data points represent the best-estimate TRA (%) from two individual feeds, with error represented as 95% CI. The original SMFA results in each feed are shown in Table S5.

(D) Kinetics data of TB31F Fab (wheat) and RUPA-117 Fab (olive) binding to hyperglycosylated Pfs48/45-6C variants. Schematic of 6C.mAgE2, 6C.mAgE2+4Gly, and 6C.mAgE2+6Gly are shown with the adjacent TB31F and RUPA-117 epitopes colored in hatched beige and olive, respectively. Red stars denote areas of potential steric clashes for RUPA-117 binding.

See also Figure S5.

mAb TB31F by >25°C through a combination of filling hydrophobic pockets, introducing a new H-bond, and releasing steric strain. The higher stability achieved by our engineered Pfs48/45-6C constructs drastically improved transmission-blocking activity, compared with WT antigen, and led to polyclonal responses in mice with TRA IC_{80}s of ~40 µg/mL, approaching the exquisite potency of transmission-blocking mAb, TB31F, which has an IC_{80} of ~1 µg/mL. This is especially noteworthy considering that the polyclonal antibodies were not affinity-purified, and therefore only a fraction of the antibodies are antigen-specific. This potency starkly contrasts with immune responses elicited by previously described Pfs48/45 constructs, which have demonstrated much lower and highly variable TRA across different studies and platforms, often with incomplete parasite inhibition even at ~750 µg/mL of purified polyclonal IgG or ~1/9 sera dilution (Singh et al., 2015, 2017, 2019; Lee et al., 2020). Our study thus provides an example of the transformative impact of structure-based immunogen design in the context of a parasitic vaccine target.

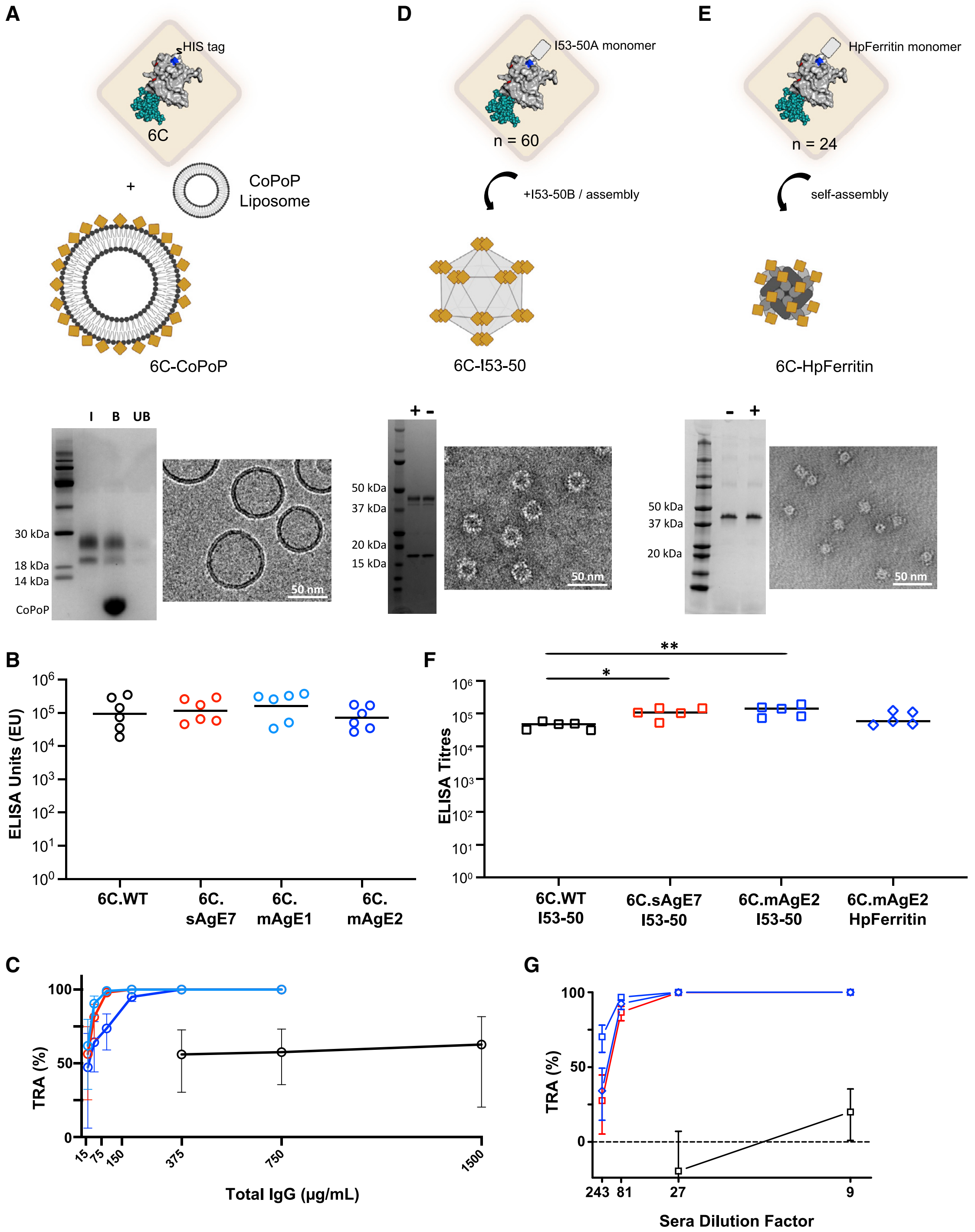

Figure 5. Pfs48/45-6C stabilized variants elicit responses associated with robust TRA across vaccine platforms

(A, D, and E) Schematic of (A) Pfs48/45-6C-CoPoP immunogens, (D) Pfs48/45-6C-I53-50 immunogens, and (E) Pfs48/45-6C-HpFerritin immunogens. The Pfs48/45-6C antigen is represented by a yellow diamond and colored as in Figure 3C. SDS-polyacrylamide gel electrophoresis (SDS-PAGE) and electron microscopy images of each multimerized 6C.mAgE2 immunogen are below its respective schematic (cryo-EM for 6C.mAgE2-CoPoP and negative-stain EM for 6C.mAgE2-I53-50 and 6C.mAgE2-HpFerritin), with "−" and "+" referring to non-reducing and reducing conditions, respectively, and I, B, and UB referring to input, bound, and unbound fractions of sample.

(B) ELISA units using 6C.sAgE7 as coated antigen for sera from immunization with stabilized Pfs48/45-6C variants formulated in CPQ. Data are plotted as individual mice (n = 6), with the bars representing the geometric mean.

(C) SMFA of serially diluted purified IgG derived from immunizations with CPQ-formulated 6C.WT (black), 6C.sAgE7 (red), 6C.mAgE1 (pale blue), and 6C.mAgE2 (blue). The best-estimate TRA (%) and 95% CI from multiple feeds are shown. The original SMFA results of each feed are in Tables S6 and S7.

(legend continued on next page)

Given that the exact biological function of Pfs48/45, and particularly the role of its 6C domain, remains poorly understood, why stabilization of this domain in this conformation leads to such drastically improved functional immune responses is not understood mechanistically at this time. Notably, as the vaccine-elicited titers did not considerably improve between the WT and engineered constructs, we conclude that the overall stabilization led to a substantial improvement in elicited antibody quality. Future work in gametocyte parasitology combined with structural biology on native Pfs48/45 and detailed characterization of the polyclonal antibody response will help define the mechanisms by which such potent parasite inhibition occurs. Nevertheless, our results showcase how antibody research, such as identifying the most potent mAbs, combined with high-resolution structural biology and structure-based design, can lead to improved immunogens capable of eliciting potent inhibitory responses—even in the absence of a complete understanding of the biological function for a target.

Here, we also investigated whether only the highly potent TB31F epitope or an extended antigenic surface on Pfs48/45-6C was crucial to elicit the most potent polyclonal antibody response. As sera elicited by hyperglycosylated 6C.mAgE2 constructs were substantially less potent in TRA, compared with the sera elicited by stabilized but non-hyperglycosylated immunogens, our data indicate that additional potent epitopes are present on Pfs48/45-6C beyond the TB31F epitope. Indeed, emerging data on human mAbs elicited during natural malaria infection identified that an antigenic surface adjacent to the TB31F epitope is also associated with recognition by potent antibodies (Fabra-García et al., 2021). Our Pfs48/45-6C stabilization efforts emphasize the utility of whole-domain stabilization of antigens instead of more restricted immunogen design strategies, such as epitope grafting (Correia et al., 2014), particularly in the absence of knowledge derived from numerous inhibitory and non-inhibitory antibodies. Such a deep structure-function understanding of antibody recognition would be required to unlock the full spectrum of structure-based immunogen design approaches, and much remains to be uncovered in this regard for essential malaria antigens across parasite life stages (Julien and Wardemann, 2019).

Beyond antigen stabilization, vaccine formulation—both in terms of multivalent antigen display and adjuvant selection—is a major component of subunit vaccine design. Several display platforms exist and have been used for TBV antigen presentation, including genetic fusions and affinity linkages (Brod et al., 2018; Chichester et al., 2018; Mabrouk et al., 2020; Scaria et al., 2020, 2021; Jardine et al., 2013; Huang et al., 2018, 2020; Mabrouk et al., 2021). An advantage of the CPQ platform used in the present study is its ability to easily incorporate antigenic designs along with adjuvants for rapid immunogenicity screening. In this study, engineered constructs emerging from expression and biochemical characterization were directly associated on cobalt-containing liposomes via their His_{6x}-tag and quickly evaluated iteratively *in vivo* to characterize the elicited immune responses, leveraging both multimerization and self-adjuvantation from the lipid content simultaneously as mechanisms to augment the immune response (Huang et al., 2018). We observed that antigenic display on diverse multimerization platforms provided an overall stronger antibody response across all malarial immunogens tested in this study, highlighting the importance of particulate display in vaccine design. The potent polyclonal antibody response achieved here, independent of delivery platforms (i.e., across liposomes and two different protein nanocages with adjuvant), demonstrates that the quality of the antigen drives the quality of the immune response. The versatility of our stabilized antigens opens the door for optimizing methods of delivery, antigen display, and adjuvantation for advancing this promising engineered TBV immunogen toward evaluation in humans. Importantly, our engineered Pfs48/45 immunogen capable of eliciting exquisite inhibitory responses also enables future studies exploring combinations with leading immunogens across Pf life-stages, such as anti-infective PfCSP-based vaccines, to deliver a multi-component malaria vaccine capable of achieving robust and durable malaria control and elimination.

Limitations of the study

The immunogenicity and functional data reported in this study are from murine immunizations and do not necessarily reflect the response that will be generated in a human IgG repertoire. Further studies are required to assess the degree to which the Pfs48/45 antigen stabilization described here will impart similar improvements to transmission-blocking activity in a human context. The SMFA experiments have an intrinsically large error, with low confidence in precise TRAs below 80% TRA without high replicates. Efforts were taken to reduce this effect via multiple replicate feeds, replicates with samples from separate immunizations, and transparent reporting of the data with full SMFA results (such as oocysts counts) included as supplemental information.

STAR★METHODS

Detailed methods are provided in the online version of this paper and include the following:

- KEY RESOURCES TABLE
- RESOURCE AVAILABILITY
  - Lead contact
  - Materials availability
  - Data and code availability
- EXPERIMENTAL MODEL AND SUBJECT DETAILS
  - Mammalian cell lines and culture conditions

(F) Immunogenicity of Pfs48/45-6C variants fused to I53-50 and HpFerritin nanocages formulated in AddaVax as measured by ELISA titers against 6C.sAgE7-coated antigen. * and ** indicate p values of ≤ 0.05, or ≤ 0.01, respectively, as determined by one-way ANOVA for log-transformed ELISA titers. ELISA measurements for Figures 5B and 5F were performed in two different laboratories using separate methods; thus, ELISA units and ELISA titers are reported distinctly.
(G) SMFA with diluted pooled sera derived from mice immunized with 6C.WT, 6C.sAgE7, and 6C.mAgE2 fusions to I53-50, and 6C.mAgE2 fusion to HpFerritin, with samples colored as in (F). Values are calculated estimates from two independent SMFA experiments, and error bars indicate 95% confidence intervals. The original SMFA results of each feed are in Table S9 and calculated TRA estimates and p values in Table S10.
See also Figure S6.

  - Mice
- METHOD DETAILS
  - *In silico* modeling of stabilized Pfs48/45-6C constructs
  - Recombinant protein expression and purification
  - Co-crystallization and structure determination
  - Hydrogen/Deuterium Exchange Mass Spectrometry
  - Plasmid construction of Pfs48/45-6C-I53-50A
  - Transient transfection of Pfs48/45-6C-I53-50A
  - Protein purification of Pfs48/45-6C-I53-50A
  - Liposome preparation and characterization
  - Microbial protein expression and purification
  - Expression and purification of Pfs48/45-6C-HpFerritin nanoparticle fusions
  - In vitro nanoparticle assembly and non-assembling control preparation
  - Endotoxin measurements for Pfs48/45-6C-I53-50 particles and controls
  - UV/vis for Pfs48/45-6C-I53-50 particles and controls
  - Biolayer interferometry for Pfs48/45-6C variants
  - Biolayer interferometry for Pfs48/45-6C-I53-50 particles and controls
  - Circular dichroism
  - Dynamic light scattering for extended thermostability analysis
  - Dynamic light scattering for Pfs48/45-6C-I53-50 particles and controls
  - Electron microscopy of CoPoP liposomes
  - Negative stain electron microscopy for Pfs48/45-6C-I53-50 particles and controls
  - Animal immunizations with Pfs48/45-6C and Pfs48/45-6C-CoPoP
  - Animal Immunizations of Pfs48/45-6C nanoparticles and controls
  - ELISA and SMFA of anti-Pfs48/45 antibodies elicited by Pfs48/45-6C and Pfs48/45-6C-CoPoP at Laboratory of Malaria and Vector Research (LMVR)
  - ELISA of antibodies elicited by Pfs48/45-6C nanoparticles and controls
  - SMFA at Radboud University Medical Center (RUMC)
- QUANTIFICATION AND STATISTICAL ANALYSIS

SUPPLEMENTAL INFORMATION

Supplemental information can be found online at https://doi.org/10.1016/j.immuni.2022.07.015.

ACKNOWLEDGMENTS

We thank Marcos Miranda for electron microscopy of *in vitro*-assembled Pfs48/45-6C-I53-50 particles, Rianne Stoter and Wiebe Kooijman for conducting SMFA experiments at RUMC, and John Rubinstein for assistance with cryo-EM. This work was supported by a National Institutes of Health grant (1R01AI148557-01A1 to J.F.L. and J.-P.J.); Bill and Melinda Gates Foundation grants (OPP1156262 to N.P.K. and J.-P.J.; and OPP1126258 to K.K.L.); a Canadian Institutes of Health Research Project grant (428410 to J.-P.J.); and in part, thanks to funding from the Bill and Melinda Gates Foundation (OPP1179906 to J.-P.J.), PATH's Malaria Vaccine Initiative under grant OPP1108403 from the Bill & Melinda Gates Foundation, the CIFAR Azrieli Global Scholar program (J.-P.J.), the Ontario Early Researcher Award program (J.-P.J.), and the Canada Research Chair program (J.-P.J.). M.J. is supported by the Netherlands Organization for Scientific Research (Vidi fellowship NWO project number 192.061). B.M. and I.K. are supported by a Hospital for Sick Children Restracomp Graduate Fellowship and Postdoctoral Fellowship, respectively, and S.H. is supported by an Ontario Graduate Scholarship program and a University of Toronto Biochemistry fellowship. The study conducted at LMVR was supported by the intramural program of the National Institute of Allergy and Infectious Disease/NIH. BLI, DLS, and CD instruments were accessed at the Structural and Biophysical Core Facility, and access to electron microscopes was through the Nanoscale Biomedical Imaging Facility at the Hospital for Sick Children and supported by the Canada Foundation for Innovation and Ontario Research Fund. X-ray diffraction experiments were performed at GM/CA@APS, which has been funded in whole or in part with federal funds from the National Cancer Institute (ACB-12002) and the National Institute of General Medical Sciences (AGM-12006). The Eiger 16M detector was funded by an NIH-Office of Research Infrastructure Programs High-End Instrumentation grant (1S10OD012289-01A1). This research used resources of the Advanced Photon Source, a U.S. Department of Energy (DOE) Office of Science user facility operated for the DOE Office of Science by Argonne National Laboratory under contract DE-AC02-06CH11357.

AUTHOR CONTRIBUTIONS

Experimental design was collaborative between all co-authors. Experiments were conducted by B.M., M.T.M., K.M., R.R., S.K., S.H., T.P.P., A.S., I.K., P.K., W.-C.H., M.J., A.B., D.P., M.M., J.C.K., E.M.L., Y.J., M.v.d.V.-B., G.-J.v.G., and J.R. The manuscript was written by B.M. and J.-P.J., and edited by all co-authors. Funding was secured by C.R.K., R.S.M., Y.W., K.K.L., M.M.J., N.P.K., J.F.L., and J.-P.J.

DECLARATION OF INTERESTS

The authors declare no competing interests. A patent application has been filed that relates to this work.

Received: May 12, 2022
Revised: July 7, 2022
Accepted: July 18, 2022
Published: August 16, 2022

REFERENCES

Acquah, F.K., Adjah, J., Williamson, K.C., and Amoah, L.E. (2019). Transmission-blocking vaccines: old friends and new prospects. Infect. Immun. *87*. e00775–e00718.

Acquah, F.K., Obboh, E.K., Asare, K., Boampong, J.N., Nuvor, S.V., Singh, S.K., Theisen, M., Williamson, K.C., and Amoah, L.E. (2017). Antibody responses to two new Lactococcus lactis-produced recombinant Pfs48/45 and Pfs230 proteins increase with age in malaria patients living in the Central Region of Ghana. Malar. J. *16*, 306.

Adams, P.D., Afonine, P.V., Bunkóczi, G., Chen, V.B., Davis, I.W., Echols, N., Headd, J.J., Hung, L.W., Kapral, G.J., Grosse-Kunstleve, R.W., et al. (2010). Phenix: a comprehensive Python-based system for macromolecular structure solution. Acta Crystallogr. D Biol. Crystallogr. *66*, 213–221.

Alexander, J., Sidney, J., Southwood, S., Ruppert, J., Oseroff, C., Maewal, A., Snoke, K., Serra, H.M., Kubo, R.T., and Sette, A. (1994). Development of high potency universal DR-restricted helper epitopes by modification of high affinity DR-blocking peptides. Immunity *1*, 751–761.

Brod, F., Miura, K., Taylor, I., Li, Y., Marini, A., Salman, A.M., Spencer, A.J., Long, C.A., and Biswas, S. (2018). Combination of RTS,S and Pfs25-IMX313 induces a functional antibody response against malaria infection and transmission in mice. Front. Immunol. *9*, 2780.

Brouwer, P.J.M., Antanasijevic, A., Berndsen, Z., Yasmeen, A., Fiala, B., Bijl, T.P.L., Bontjer, I., Bale, J.B., Sheffler, W., Allen, J.D., et al. (2019). Enhancing and shaping the immunogenicity of native-like HIV-1 envelope trimers with a two-component protein nanoparticle. Nat. Commun. *10*, 4272.

Chemical Computing Group (2022). Molecular Operating Environment (MOE). Chemical Computing Group ULC, 1010 Sherbooke St. West, Suite #910, Montreal, QC, Canada, H3A 2R7, 2022.

CellPress
OPEN ACCESS

Chichester, J.A., Green, B.J., Jones, R.M., Shoji, Y., Miura, K., Long, C.A., Lee, C.K., Ockenhouse, C.F., Morin, M.J., Streatfield, S.J., et al. (2018). Safety and immunogenicity of a plant-produced Pfs25 virus-like particle as a transmission blocking vaccine against malaria: a phase 1 dose-escalation study in healthy adults. Vaccine *36*, 5865–5871.

Coelho, C.H., Tang, W.K., Burkhardt, M., Galson, J.D., Muratova, O., Salinas, N.D., Alves E Silva, T.L., Reiter, K., MacDonald, N.J., Nguyen, V., et al. (2021). A human monoclonal antibody blocks malaria transmission and defines a highly conserved neutralizing epitope on gametes. Nat. Commun. *12*, 1750.

Corbett, K.S., Edwards, D.K.,, Leist, S.R., Abiona, O.M., Boyoglu-Barnum, S., Gillespie, R.A., Himansu, S., Schäfer, A., Ziwawo, C.T., DiPiazza, A.T., et al. (2020). SARS-CoV-2 mRNA vaccine design enabled by prototype pathogen preparedness. Nature *586*, 567–571.

Correia, B.E., Bates, J.T., Loomis, R.J., Baneyx, G., Carrico, C., Jardine, J.G., Rupert, P., Correnti, C., Kalyuzhniy, O., Vittal, V., et al. (2014). Proof of principle for epitope-focused vaccine design. Nature *507*, 201–206.

Das, R., and Baker, D. (2008). Macromolecular modeling with Rosetta. Annu. Rev. Biochem. *77*, 363–382.

Delves, M.J., Angrisano, F., and Blagborough, A.M. (2018). Antimalarial transmission-blocking interventions: past, present, and future. Trends Parasitol. *34*, 735–746.

Duffy, P.E., and Patrick Gorres, J. (2020). Malaria vaccines since 2000: progress, priorities, products. npj Vaccines *5*, 48.

Emsley, P., Lohkamp, B., Scott, W.G., and Cowtan, K. (2010). Features and development of coot. Acta Crystallogr. D Biol. Crystallogr. *66*, 486–501.

Fabra-García, A., et al. (2021). Potent Human Monoclonal Antibodies Target Different Domains of Malaria Transmission Blocking Vaccine Candidate Pfs48/45 (American Society of Tropical Medicine and Hygiene). nr 1166.

Florens, L., Washburn, M.P., Raine, J.D., Anthony, R.M., Grainger, M., Haynes, J.D., Moch, J.K., Muster, N., Sacci, J.B., Tabb, D.L., et al. (2002). A proteomic view of the Plasmodium falciparum life cycle. Nature *419*, 520–526.

Gasteiger, E., Hoogland, C., Gattiker, A., Duvaud, S.'., Wilkins, M.R., Appel, R.D., and Bairoch, A. (2005). Protein identification and analysis tools on the Expasy server. In The Proteomics Protocols Handbook (Humana Press), pp. 571–607.

Guttman, M., Weis, D.D., Engen, J.R., and Lee, K.K. (2013). Analysis of overlapped and noisy hydrogen/deuterium exchange mass spectra. J. Am. Soc. Mass Spectrom. *24*, 1906–1912.

Healy, S.A., Anderson, C., Swihart, B.J., Mwakingwe, A., Gabriel, E.E., Decederfelt, H., Hobbs, C.V., Rausch, K.M., Zhu, D., Muratova, O., et al. (2021). Pfs230 yields higher malaria transmission-blocking vaccine activity than Pfs25 in humans but not mice. J. Clin. Invest. *131*, e146221.

Hsieh, C.L., Goldsmith, J.A., Schaub, J.M., DiVenere, A.M., Kuo, H.C., Javanmardi, K., Le, K.C., Wrapp, D., Lee, A.G., Liu, Y., et al. (2020). Structure-based design of prefusion-stabilized SARS-CoV-2 spikes. Science *369*, 1501–1505.

Huang, W.C., Deng, B., Lin, C., Carter, K.A., Geng, J., Razi, A., He, X., Chitgupi, U., Federizon, J., Sun, B., et al. (2018). A malaria vaccine adjuvant based on recombinant antigen binding to liposomes. Nat. Nanotechnol. *13*, 1174–1181.

Huang, W.C., Deng, B., Seffouh, A., Ortega, J., Long, C.A., Suresh, R.V., He, X., Miura, K., Lee, S.M., Wu, Y., et al. (2020). Antibody response of a particle-inducing, liposome vaccine adjuvant admixed with a Pfs230 fragment. npj Vaccines *5*, 23.

Jardine, J., Julien, J.P., Menis, S., Ota, T., Kalyuzhniy, O., McGuire, A., Sok, D., Huang, P.S., MacPherson, S., Jones, M., et al. (2013). Rational HIV immunogen design to target specific germline B cell receptors. Science *340*, 711–716.

Julien, J.P., and Wardemann, H. (2019). Antibodies against Plasmodium falciparum malaria at the molecular level. Nat. Rev. Immunol. *19*, 761–775.

Kabsch, W. (2010). XDS. Acta Crystallogr. D Biol. Crystallogr. *66*, 125–132.

Kundu, P., Semesi, A., Jore, M.M., Morin, M.J., Price, V.L., Liang, A., Li, J., Miura, K., Sauerwein, R.W., King, C.R., et al. (2018). Structural delineation of potent transmission-blocking epitope I on malaria antigen Pfs48/45. Nat. Commun. *9*, 4458.

Lee, S.-M., Hickey, J.M., Miura, K., Joshi, S.B., Volkin, D.B., King, C.R., and Plieskatt, J.L. (2020). A C-terminal Pfs48/45 malaria transmission-blocking vaccine candidate produced in the baculovirus expression system. Sci. Rep. *10*, 395.

Mabrouk, M.T., Huang, W.C., Deng, B., Li-Purcell, N., Seffouh, A., Ortega, J., Ekin Atilla-Gokcumen, G., Long, C.A., Miura, K., and Lovell, J.F. (2020). Lyophilized, antigen-bound liposomes with reduced MPLA and enhanced thermostability. Int. J. Pharm. *589*, 119843.

Mabrouk, M.T., Chiem, K., Rujas, E., Huang, W.C., Jahagirdar, D., Quinn, B., Surendran Nair, M., Nissly, R.H., Cavener, V.S., Boyle, N.R., et al. (2021). Lyophilized, thermostable Spike or RBD immunogenic liposomes induce protective immunity against SARS-CoV-2 in mice. Sci. Adv. *7*, 1476.

Marr, C.R., Benlekbir, S., and Rubinstein, J.L. (2014). Fabrication of carbon films with ~ 500nm holes for cryo-EM with a direct detector device. J. Struct. Biol. *185*, 42–47.

McCoy, A.J., Grosse-Kunstleve, R.W., Adams, P.D., Winn, M.D., Storoni, L.C., and Read, R.J. (2007). Phaser crystallographic software. J. Appl. Crystallogr. *40*, 658–674.

McLellan, J.S., Chen, M., Joyce, M.G., Sastry, M., Stewart-Jones, G.B., Yang, Y., Zhang, B., Chen, L., Srivatsan, S., Zheng, A., et al. (2013). Structure-based design of a fusion glycoprotein vaccine for respiratory syncytial virus. Science *342*, 592–598.

McLeod, B., Miura, K., Scally, S.W., Bosch, A., Nguyen, N., Shin, H., Kim, D., Volkmuth, W., Rämisch, S., Chichester, J.A., et al. (2019). Potent antibody lineage against malaria transmission elicited by human vaccination with Pfs25. Nat. Commun. *10*, 4328.

Miura, K., Deng, B., Wu, Y., Zhou, L., Pham, T.P., Diouf, A., Wu, C.K., Lee, S.M., Plieskatt, J.L., Morin, M.J., et al. (2019). ELISA units, IgG subclass ratio and avidity determined functional activity of mouse anti-Pfs230 antibodies judged by a standard membrane-feeding assay with Plasmodium falciparum. Vaccine *37*, 2073–2078.

Miura, K., Orcutt, A.C., Muratova, O.V., Miller, L.H., Saul, A., and Long, C.A. (2008). Development and characterization of a standardized ELISA including a reference serum on each plate to detect antibodies induced by experimental malaria vaccines. Vaccine *26*, 193–200.

Miura, K., Swihart, B.J., Deng, B., Zhou, L., Pham, T.P., Diouf, A., Burton, T., Fay, M.P., and Long, C.A. (2016). Transmission-blocking activity is determined by transmission-reducing activity and number of control oocysts in Plasmodium falciparum standard membrane-feeding assay. Vaccine *34*, 4145–4151.

Miura, K., Deng, B., Tullo, G., Diouf, A., Moretz, S.E., Locke, E., Morin, M., Fay, M.P., and Long, C.A. (2013). Qualification of standard membrane-feeding assay with plasmodium falciparum malaria and potential improvements for future assays. PLoS one *8*, e57909.

Morin, A., Eisenbraun, B., Key, J., Sanschagrin, P.C., Timony, M.A., Ottaviano, M., and Sliz, P. (2013). Collaboration gets the most out of software. eLife *2*, e01456.

Outchkourov, N.S., Roeffen, W., Kaan, A., Jansen, J., Luty, A., Schuiffel, D., van Gemert, G.J., van de Vegte-Bolmer, M., Sauerwein, R.W., and Stunnenberg, H.G. (2008). Correctly folded Pfs48/45 protein of Plasmodium falciparum elicits malaria transmission-blocking immunity in mice. Proc. Natl. Acad. Sci. USA *105*, 4301–4305.

PAHO/WHO|Pan American Health Organization (2021). Technical Note: malaria vaccine and priorities for malaria elimination in the Americas. https://www.paho.org/en/documents/technical-note-malaria-vaccine-and-priorities-malaria-elimination-americas.

Pallesen, J., Wang, N., Corbett, K.S., Wrapp, D., Kirchdoerfer, R.N., Turner, H.L., Cottrell, C.A., Becker, M.M., Wang, L., Shi, W., et al. (2017). Immunogenicity and structures of a rationally designed prefusion MERS-CoV spike antigen. Proc. Natl. Acad. Sci. USA *114*, E7348–E7357.

Ponnudurai, T., Lensen, A.H., Van Gemert, G.J., Bensink, M.P., Bolmer, M., and Meuwissen, J.H. (1989). Infectivity of cultured Plasmodium falciparum gametocytes to mosquitoes. Parasitology *98*, 165–173.

Scaria, P.V., Anderson, C., Muratova, O., Alani, N., Trinh, H.V., Nadakal, S.T., Zaidi, I., Lambert, L., Beck, Z., Barnafo, E.K., et al. (2021). Malaria transmission-blocking conjugate vaccine in ALFQ adjuvant induces durable functional immune responses in rhesus macaques. npj Vaccines *6*, 148.

Scaria, P.V., Chen, B.B., Rowe, C.G., Alani, N., Muratova, O.V., Barnafo, E.K., Lambert, L.E., Zaidi, I.U., Lees, A., Rausch, K.M., et al. (2020). Comparison of carrier proteins to conjugate malaria transmission blocking vaccine antigens, Pfs25 and Pfs230. Vaccine *38*, 5480–5489.

Sherrard-Smith, E., Sala, K.A., Betancourt, M., Upton, L.M., Angrisano, F., Morin, M.J., Ghani, A.C., Churcher, T.S., and Blagborough, A.M. (2018). Synergy in anti-malarial pre-erythrocytic and transmission-blocking antibodies is achieved by reducing parasite density. eLife *7*, e35213.

Singh, S.K., Roeffen, W., Andersen, G., Bousema, T., Christiansen, M., Sauerwein, R., and Theisen, M. (2015). A *Plasmodium falciparum* 48/45 single epitope R0.6C subunit protein elicits high levels of transmission blocking antibodies. Vaccine *33*, 1981–1986.

Singh, S.K., Thrane, S., Janitzek, C.M., Nielsen, M.A., Theander, T.G., Theisen, M., Salanti, A., and Sander, A.F. (2017). Improving the malaria transmission-blocking activity of a Plasmodium falciparum 48/45 based vaccine antigen by SpyTag/SpyCatcher mediated virus-like display. Vaccine *35*, 3726–3732.

Singh, S.K., Thrane, S., Chourasia, B.K., Teelen, K., Graumans, W., Stoter, R., van Gemert, G.J., van de Vegte-Bolmer, M.G., Nielsen, M.A., Salanti, A., et al. (2019). Pfs230 and Pfs48/45 fusion proteins elicit strong transmission-blocking antibody responses against plasmodium falciparum. Front. Immunol. *10*, 1256.

Singh, S.K., Plieskatt, J., Chourasia, B.K., Fabra-García, A., Garcia-Senosiain, A., Singh, V., Bengtsson, K.L., Reimer, J.M., Sauerwein, R., Jore, M.M., and Theisen, M. (2021). A reproducible and scalable process for manufacturing a Pfs48/45 based *Plasmodium falciparum* transmission-blocking vaccine. Front. Immunol. *11*, 3369.

Stone, W.J.R., Campo, J.J., Ouédraogo, A.L., Meerstein-Kessel, L., Morlais, I., Da, D., Cohuet, A., Nsango, S., Sutherland, C.J., van de Vegte-Bolmer, M., et al. (2018). Unravelling the immune signature of *Plasmodium falciparum* transmission-reducing immunity. Nat. Commun. *9*, 558.

Stone, W.J.R., Dantzler, K.W., Nilsson, S.K., Drakeley, C.J., Marti, M., Bousema, T., and Rijpma, S.R. (2016). Naturally acquired immunity to sexual stage *P. falciparum* parasites. Parasitology *143*, 187–198.

Talapko, J., Škrlec, I., Alebić, T., Jukić, M., and Včev, A. (2019). Malaria: the past and the present. Microorganisms *7*, 179.

The RTS S Clinical Trials Partnership (2012). A Phase 3 trial of RTS,S/AS01 malaria vaccine in African infants. N. Engl. J. Med. *367*, 2284–2295.

Tivol, W.F., Briegel, A., and Jensen, G.J. (2008). An improved cryogen for plunge freezing. Microsc. Microanal. *14*, 375–379.

van der Boor, S.C., et al. (2020). First-in-Human Evaluation of a *Plasmodium falciparum* Transmission-Blocking Monoclonal Antibody (American Society of Tropical Medicine and Hygiene). virtual (2020), nr 678.

van Dijk, M.R., Janse, C.J., Thompson, J., Waters, A.P., Braks, J.A., Dodemont, H.J., Stunnenberg, H.G., van Gemert, G.J., Sauerwein, R.W., and Eling, W. (2001). A central role for P48/45 in malaria parasite male gamete fertility. Cell *104*, 153–164.

Verkerke, H.P., Williams, J.A., Guttman, M., Simonich, C.A., Liang, Y., Filipavicius, M., Hu, S.L., Overbaugh, J., and Lee, K.K. (2016). Epitope-independent purification of native-like envelope trimers from diverse HIV-1 isolates. J. Virol. *90*, 9471–9482.

Weis, D.D., Engen, J.R., and Kass, I.J. (2006). Semi-automated data processing of hydrogen exchange mass spectra using HX-Express. J. Am. Soc. Mass Spectrom. *17*, 1700–1703.

White, M.T., Verity, R., Griffin, J.T., Asante, K.P., Owusu-Agyei, S., Greenwood, B., Drakeley, C., Gesase, S., Lusingu, J., Ansong, D., et al. (2015). Immunogenicity of the RTS,S/AS01 malaria vaccine and implications for duration of vaccine efficacy: secondary analysis of data from a phase 3 randomised controlled trial. Lancet Infect. Dis. *15*, 1450–1458.

World Health Organization (2021). World malaria report 2021. https://www.who.int/teams/global-malaria-programme/reports/world-malaria-report-2021.

Wuertz, K.M.G., Barkei, E.K., Chen, W.H., Martinez, E.J., Lakhal-Naouar, I., Jagodzinski, L.L., Paquin-Proulx, D., Gromowski, G.D., Swafford, I., Ganesh, A., et al. (2021). A SARS-CoV-2 spike ferritin nanoparticle vaccine protects hamsters against Alpha and Beta virus variant challenge. npj Vaccines *6*, 129.

STAR★METHODS

KEY RESOURCES TABLE

REAGENT or RESOURCE	SOURCE	IDENTIFIER
Antibodies		
TB31F	Kundu et al. (2018)	N/A
RUPA-47	This paper	N/A
RUPA-117	This paper	N/A
anti-mouse horseradish peroxidase-conjugated horse IgG	Cell Signaling Technology	Cat#7076S
Chemicals, peptides, and recombinant proteins		
6C.WT	Kundu et al. (2018)	N/A
6C.sAgE1	This paper	N/A
6C.sAgE2	This paper	N/A
6C.sAgE3	This paper	N/A
6C.sAgE4	This paper	N/A
6C.sAgE5	This paper	N/A
6C.sAgE6	This paper	N/A
6C.sAgE7	This paper	N/A
6C.mAgE1	This paper	N/A
6C.mAgE2	This paper	N/A
6C.TB-KO	This paper	N/A
6C.mAgE2+4Gly	This paper	N/A
6C.mAgE2+6Gly	This paper	N/A
CoPoP Liposomes	Huang et al. (2018)	N/A
I53-50B	Brouwer et al. (2019)	N/A
Pfs48/45-6C.sAgE7-I53-50A	This paper	N/A
Pfs48/45-6C.mAgE1-I53-50A	This paper	N/A
Pfs48/45-6C.mAgE2-I53-50A	This paper	N/A
GIBCO™ FreeStyle™ 293 Expression Medium	Thermo Fisher Scientific	Cat#12338026
FectoPRO DNA Transfection Reagent	VWR	Cat#10118-444
PrestoBlue Cell Viability Reagent	Thermo Fisher Scientific	Cat#A13262
PEI-MAX	Polysciences	Cat# 24765-1
Poly(diallyldimethylammonium chloride) solution	Sigma-Aldrich	Cat#409014
AddaVax	InvivoGen	Cat# vac-adx-10
TMB	SeraCare	Cat#5120-0047
Critical commercial assays		
Ni-NTA biosensors	ForteBio	Cat#18-5102
Anti-human Fab-CH1 biosensors	ForteBio	Cat#18-5125
Anti-penta-HIS biosensors	Sartorius	Cat# 18-5120
10 well 4-20% gradient SDS-PAGE gel	Bio-Rad	Cat#4561096
10x Tris/Glycine/SDS Buffer	Bio-Rad	Cat#1610732
Precision Plus Dual Colour Standards	Bio-Rad	Cat#1610394
Novex 4-12% bis-tris acrylamide gels	Invitrogen	Cat#NP0321BOX
MES Running Buffer	Invitrogen	Cat#NP0002
Deposited data		
Crystal structure of 6C.mAgE1-RUPA-47 Fab-RUPA-117 Fab complex	This paper	PDB: 7UNB

(*Continued on next page*)

Continued		
REAGENT or RESOURCE	SOURCE	IDENTIFIER
Experimental models: Cell lines		
FreeStyle^{TM} 293-F Cells	Thermo Fisher Scientific	Cat#R79007
Expi293F GnTI -/- Cells	Thermo Fisher Scientific	A39240
Lemo21(DE3)	NEB	Cat#C2528J
Experimental models: Organisms/strains		
Mouse: Crl:CD1(ICR)	Charles River Laboratories	Strain Code 022
Parasite: *P. falciparum ; NF54 strain*	Miura et al. (2013)	N/A
Mosquito: *Anopheles stephensi* (Nijmegen strain)	Ponnudurai et al. (1989)	N/A
Recombinant DNA		
pcDNA3.4_6C.WT-His	Kundu et al. (2018)	N/A
pcDNA3.4_TB31F_HC	Kundu et al. (2018)	N/A
pcDNA3.4_TB31F_LC	Kundu et al. (2018)	N/A
pcDNA3.4_RUPA-117_HC	This Paper	N/A
pcDNA3.4_RUPA-117_KC	This Paper	N/A
pcDNA3.4_RUPA-47_HC	This Paper	N/A
pcDNA3.4_RUPA-47_KC	This Paper	N/A
pcDNA3.4_6C.sAgE1-His	This Paper	N/A
pcDNA3.4_6C.sAgE2-His	This Paper	N/A
pcDNA3.4_6C.sAgE3-His	This Paper	N/A
pcDNA3.4_6C.sAgE4-His	This Paper	N/A
pcDNA3.4_6C.sAgE5-His	This Paper	N/A
pcDNA3.4_6C.sAgE6-His	This Paper	N/A
pcDNA3.4_6C.sAgE7-His	This Paper	N/A
pcDNA3.4_6C.mAgE1-His	This Paper	N/A
pcDNA3.4_6C.mAgE2-His	This Paper	N/A
pcDNA3.4_6C.TB-KO-His	This paper	N/A
pcDNA3.4_6C.mAgE2+4Gly-His	This paper	N/A
pcDNA3.4_6C.mAgE2+6Gly-His	This paper	N/A
I53-50B	Brouwer et al. (2019)	N/A
Pfs48/45-6C.sAgE7-I53-50A	This paper	N/A
Pfs48/45-6C.mAgE1-I53-50A	This paper	N/A
Pfs48/45-6C.mAgE2-I53-50A	This paper	N/A
Software and algorithms		
Rosetta	Das and Baker (2008)	https://www.rosettacommons.org/software
Phenix	Adams et al. (2010)	http://www.phenix-online.org/
XDS	Kabsch (2010)	https://xds.mr.mpg.de/html_doc/downloading.html
Coot	Emsley et al. (2010)	https://www2.mrc-lmb.cam.ac.uk/personal/pemsley/coot/
PRISM Graphpad	GraphPad Software, LLC	https://www.graphpad.com/scientific-software/prism/
Molecular Operating Environment	Chemical Computing Group, LLC	https://www.chemcomp.com/index.htm
Other		
Ni-NTA Magnetic Beads	Thermo Fischer Scientific	Cat#88831
Magnetic Separator	Thermo Fischer Scientific	Cat#12321D
Homemade holey gold grids	Marr et al. (2014)	N/A

CellPress
OPEN ACCESS

RESOURCE AVAILABILITY

Lead contact

Further information and requests for resources and reagents should be directed to and will be fulfilled by the lead contact, Jean-Philippe Julien (jean-philippe.julien@sickkids.ca).

Materials availability

All unique and stable reagents generated in this study are available via the lead contact upon a reasonable request.

Data and code availability

- The crystal structure has been deposited to the Protein Data Bank and is publicly available as of the date of publication. Accession numbers are listed in the key resources table.
- This paper does not report original code.
- Any additional information required to reanalyze the data reported in this paper is available from the lead contact upon request.

EXPERIMENTAL MODEL AND SUBJECT DETAILS

Mammalian cell lines and culture conditions

For recombinant protein expression, female mammalian cells (HEK 293F, FreeStyle™ 293-F cells, Thermo Fisher Scientific; HEK 293S, GnT $I^{-/-}$ cells, ATCC) were cultured in suspension in GIBCO™ FreeStyle™ 293 Expression Medium (Thermo Fisher Scientific) for 5-7 days at 37°C, with 70% humidity and 8% CO_2 and rotating at 150 RPM.

Mice

Naïve female ICR mice were maintained in a specific pathogen-free vivarium, housed in a climate-controlled room on a 12-hour day/night cycle, and had access to food and water *ad libitum*. Immunizations of the mice occurred between six to eight weeks of age, and groups of mice were randomly populated. Animal protocols involving these mice were approved and performed in accordance with the Institutional Animal Care and Use Committee at University at Buffalo. 8-week female Crl:CD1(ICR) mice were also used in this study and were randomly assigned across experimental groups. Animal protocols involving these mice followed the National Institute of Health's Guideline for the Care and Use of Laboratory Animals and were approved by the University of Washington's Institutional Animal Care and Use Committee.

METHOD DETAILS

In silico modeling of stabilized Pfs48/45-6C constructs

The 6CG397L model was generated by visualization of the previous Pfs48/45-6C-TB31F Fab complex crystal structure (PDB ID: 6E63). This structure was prepared using QuickPrep in MOE, and models were generated through MOE's residue scanning function (Chemical Computing Group, 2022). Predicted stability changes were determined by model generation using MOE's scoring algorithms (Chemical Computing Group, 2022). Next, the pmut function in the Rosetta Modelling suite was used to design the 6C.sAgE series of double stability mutations, by mutating every residue on the 6CG397L structure and scoring the resulting constructs by intrinsic stability (Das and Baker, 2008). The top 500 were manually sorted based on which region of the antigen they mapped to. The top five mutations from each region were analyzed by visual inspection and the most promising candidates were chosen for construct design and recombinant expression.

Recombinant protein expression and purification

All Pfs48/45-6C antigen variants were based on consensus 3D7 sequence, gene synthesized and cloned into the pcDNA3.4 expression vector (GeneArt). Antigens were transiently expressed in HEK293F cells (ThermoFisher Scientific) and purified using a 5 mL HisTrap FF column (GE Healthcare), followed by size exclusion chromatography (Superdex 200 Increase 10/300 GL, GE Healthcare). 6C.TB-KO was generated by analyzing the TB31F-6C structure (PDB ID: 6E63), and mutating a key residue involved in the antigen-antibody interaction (K416D) to knock-out TB31F binding. 6C.mAgE2+4Gly and 6C.mAgE2+6Gly were designed using visual inspection of the same structure, and inserting N-linked glycosylation sequons into regions that would not be expected to perturb TB31F binding (6C.mAgE2+4Gly: K307N, L318N, D320T, D380N, E382T, K404N; 6C.mAgE2+6Gly: K307N, L318N, D320T, L339T, E362N, L364T, D380N, E382T, K404N)

All Fabs were transiently expressed in HEK293F cells (ThermoFisher Scientific) and purified as previously described (Kundu et al., 2018; McLeod et al., 2019). Briefly, LambdaSelect or KappaSelect affinity (GE Healthcare) and size exclusion (Superdex 200 Increase 10/300 GL, GE Healthcare) chromatography columns were used; for some Fabs, cation exchange (MonoS, GE Healthcare) was also used to achieve higher purity.

Co-crystallization and structure determination

6C.mAgE1, RUPA-117 Fab, and RUPA-47 Fab were complexed at a 1:1.5:1.5 ratio to form a ternary complex. The antigen-Fab-Fab ternary complex was separated from excess Fab by size exclusion chromatography using a Superdex 200 Increase column (GE Healthcare). The resulting 6C-RUPA-117-RUPA-47 complex was concentrated to 7.5 mg/mL. Sitting-drop crystallization trays for the 6C-RUPA-47-RUPA-117 complex were set up at 2:1:2 ratio of protein complex, seeds, and reservoir solution. 6C-RUPA-117-RUPA-47 crystals grew in 0.2 M magnesium chloride, 25 % (w/v) polyethylene glycol 3350, and 0.1 M Tris, pH 8.5. Well-diffracting crystals for 6C.mAgE2 in complex with Fabs could not be obtained. Data were collected on at the 23ID beamline at the Advanced Photon Source (APS), and subsequently processed using XDS and Xprep (Kabsch, 2010). Phaser was used for molecular replacement with Pfs48/45-6C (PDB ID: 6E63) and Fabs from our internal database as search models (McCoy et al., 2007). Resulting structures were built and refined using phenix.refine (Adams et al., 2010) and Coot (Emsley et al., 2010), accessed through SBGrid (Morin et al., 2013).

Hydrogen/Deuterium Exchange Mass Spectrometry

Samples were prepared using 120 pmol (2 μg) of protein incubated in deuterated buffer (150 mM NaCl, 20 mM Na_3PO_4, 0.02% sodium azide, 85% D_2O, pH* 7.66) for 3 s, 1 min, 30 min, and 5 h at room temperature. The reaction was stopped by adding an equal volume of ice-cold quench buffer (200 mM tris)2-chorethyl) phosphate (TCEP), 0.2% formic acid) to a final pH of 2.5. Samples were immediately frozen with liquid nitrogen and stored at -80°C until analysis. Undeuterated controls were prepared similarly but using optima water in place of D_2O. The zero-time point was made by adding the protein directly to quenched deuteration buffer before freezing. Fully deuterated controls were prepared by collecting pepsin digestion eluate from two undeuterated samples. The digested peptides were concentrated under vacuum, resuspended in deuteration buffer, and heated to 65°C for one hour before they were subsequently quenched and frozen. To monitor protein stability during the longest exchange reaction, a 3 s pulse was performed alongside the 5 h time point. All exchanges were performed in triplicate.

As previously described, samples were thawed on ice and digested using an online pepsin column kept at 20°C (Verkerke et al., 2016). The peptic peptides were resolved over a Waters ACQUITY UPLC CSH C18 VanGuard, 130 Å, 1.7 μm, 1 mm by 100 mm column using a 15 min linear gradient and analyzed on a Waters Synapt G2-Si Q-TOF mass spectrometer with ion mobility enabled (Verkerke et al., 2016). Peptide assignments were made from MSE data collected for undeuterated samples using Byonic (Version 3.8, Protein Metrics Inc.). Deuterium uptake analysis was performed with HD-Examiner (Sierra Analytics) and HX-Express v2 for binomial fitting (Weis et al., 2006; Guttman et al., 2013). The percent exchange was normalized to the fully deuterated sample and corrected for in-exchange using the zero timepoint. Internal exchange standards (Pro-Pro-Pro-Ile [PPPI] and Pro-Pro-Pro-Phe [PPPF]) were included in each reaction to ensure that conditions were consistent throughout the experiment. Data for each timepoint was collected in duplicate.

Plasmid construction of Pfs48/45-6C-I53-50A

The Pfs48/45-6C variants were genetically fused to the N terminus of the trimeric I53-50A nanoparticle component using a linker of 8 glycine and serine residues. Pfs48/45-6C-8GS-I53-50A fusions were synthesized and cloned by Genscript into pCMV with an N-terminal secrecon signal peptide and a C-terminal hexa-histidine tag.

Transient transfection of Pfs48/45-6C-I53-50A

Pfs48/45-6C-I53-50A proteins were produced in Expi293F cells grown in suspension using Expi293F expression medium (Life Technologies) at 33°C, 70% humidity, 8% CO_2 rotating at 150 rpm. The cultures were transfected using PEI-MAX (Polyscience) with cells grown to a density of 3.0 million cells per mL and cultivated for 3 days. Supernatants were clarified by centrifugation (5 min at 4000 x g), the addition of PDADMAC solution to a final concentration of 0.0375% (Sigma Aldrich, #409014), and a second spin (5 min at 4000 x g).

Protein purification of Pfs48/45-6C-I53-50A

Proteins containing His tags were purified from clarified supernatants via an AKTA AVANT FPLC using prepacked 5mL Ni Sepharose excel resin columns (Cytiva), with each clarified supernatant was supplemented with 1 M Tris-HCl pH 8.0 to a final concentration of 45 mM and 5 M NaCl to a final concentration of ~310 mM, prior to column application. The resin was washed with 5 column volumes (CV) of 20 mM Tris pH 7.0, 150 mM NaCl, and the protein was eluted over 10CV column up to 100%B buffer, which was composed of 20 mM Tris pH 7.0, 150 mM NaCl, 500 mM imidazole. SDS-PAGE was used to assess the purity of peak fractions of interest. IMAC elutions were concentrated to ~2mL in a hydrated 10K molecular weight cutoff dialysis cassette (Millipore). Samples were then further purified by sizing on a Superdex S200 Increase 10/30 GL (Cytiva) in PBS + 5% glycerol. Peak fractions were pooled and analyzed by SDS-PAGE and Octet and measured for endotoxin content before freezing in single-use aliquots stored at -80°C.

Liposome preparation and characterization

CoPoP/PHAD/QS-21 liposomes had a [DOPC:CHOL:MPLA:CoPoP:QS-21] mass ratio of [20:5:0.4:1:0.4]. Liposomes were prepared as previously described using ethanol injection and nitrogen pressurized lipid extrusion in PBS, carried out at 50°C (Mabrouk et al., 2020). Ethanol was then removed by dialysis against PBS twice at 4°C. QS-21 (1 mg/mL) was added to the liposomes after formation at an equal mass ratio as PHAD. The final liposome concentration was adjusted to 320 mg/mL CoPoP, and liposomes were sterile

filtered and stored at 4°C. Liposome sizes were determined by dynamic light scattering (DLS) with a NanoBrook 90 plus PALS instrument after 200-fold dilution in PBS.

To assess the stability of constructs of interest bound in particle form, a Ni-NTA competition assay was carried out as previously described (Mabrouk et al., 2021). Ni-NTA magnetic beads (ThermoFisher Scientific, catalog no. 88831) were added to the liposome-incubated Pfs48/45 constructs (1:4 mass ratio of total protein:CoPoP) and incubated with the beads for 30 min at RT, then the supernatant and beads were separated using a magnetic separator (ThermoFisher Scientific, catalog no. 12321D). Denaturing reducing loading dye was then added to the samples (supernatant or beads) and heated at 95°C for 10 min. The samples were then subjected to SDS-polyacrylamide gel electrophoresis (SDS-PAGE) using Novex 4 to 12% bis-tris acrylamide gels (Invitrogen, catalog no. NP0321BOX) and MES running buffer (Invitrogen, catalog no. NP0002).

To determine the binding kinetics and stability of protein-liposomes, Pfs48/45 constructs were labeled with DY-490-SE (Dyomics) with a molar ratio of 5:1 dye to Pfs48/45 construct. The reaction was done in 50 mM sodium bicarbonate buffer (pH 9) and incubated with shaking for 1 h at room temperature. Free dye was removed by dialysis against PBS overnight at 4°C. Binding kinetics of labeled Pfs48/45 constructs was monitored as a function of fluorescence quenching effect due to the proximity of the dye to the porphyrin ring after binding to CoPoP liposomes but not PoP liposomes resulting in energy transfer from the dye to the porphyrin over a 3 h period. Serum stability of bound labeled constructs was tested in the same manner where after incubation for 3 h, the pre-bound protein/liposomes were transferred into 20 % serum in PBS and kept shaking at 37C over the following 24 h where the fluorescence was monitored. The binding to liposomes was calculated by the following equation:

$$\text{Pfs48/45} - \text{DY} - 490 \ \text{binding} \ \% = \left[1 - \left(\text{FI}_{\text{incubated sample}} \big/ \text{FI}_{\text{Pfs48/45}-\text{DY}-490 \text{ in PBS}}\right)\right] \times 100\%$$

The fluorescence was read at excitation/emission 480/551 nm in a microplate reader (TECAN Safire).

Microbial protein expression and purification

The I53-50B.4.PT1 and 2obx proteins were expressed in Lemo21(DE3) (NEB) in LB (10 g Tryptone, 5 g Yeast Extract, 10 g NaCl) grown in 2 L baffled shake flasks or a 10 L BioFlo 320 Fermenter (Eppendorf). Cells were grown at 37°C to an OD600 ~0.8, and then induced with 1 mM IPTG. Expression temperature was reduced to 18°C and the cells shaken for ~16 h. The cells were harvested and lysed by microfluidization using a Microfluidics M110P at 18,000 psi in 50 mM Tris, 500 mM NaCl, 30 mM imidazole, 1 mM PMSF, 0.75% CHAPS. Lysates were clarified by centrifugation at 24,000 g for 30 min and applied to a 2.6 × 10 cm Ni Sepharose 6 FF column (Cytiva) for purification by IMAC on an AKTA Avant150 FPLC system (Cytiva). Protein of interest was eluted over a linear gradient of 30 mM to 500 mM imidazole in a background of 50 mM Tris pH 8, 500 mM NaCl, 0.75% CHAPS buffer. Peak fractions were pooled, concentrated in 10K MWCO centrifugal filters (Millipore), sterile filtered (0.22 μm) and applied to either a Superdex 200 Increase 10/300, or HiLoad S200 pg GL SEC column (Cytiva) using 50 mM Tris pH 8, 500 mM NaCl, 0.75% CHAPS buffer. I53-50B.4PT1 and 2obx elute at ~0.45 CV. After sizing, bacterial-derived components were tested to confirm low levels of endotoxin before using for nanoparticle assembly.

Expression and purification of Pfs48/45-6C-HpFerritin nanoparticle fusions

Pfs48/45-6C-HpFerritin nanoparticle fusions containing the following mutations from native Hp ferritin sequence (K75N, E77T, E99N, and I101T; and addition of a PADRE sequence (Alexander et al., 1994) at the C-terminus: AKFVAAWTLKAAA) were transiently expressed in HEK293S (Gnt I$^{-/-}$) cells (ThermoFisher Scientific), and purified using a StrepTrap HP (GE Healthcare) column, followed by TEV cleavage for tag removal. Ferritin fusion nanocages were further purified to size homogeneity on a Superose 6 10/300 GL column (GE Healthcare). To verify their integrity by negative stain electron microscopy, 6C.mAgE2-HpFerritin nanoparticles were diluted to 50 μg/mL. Sample was deposited onto homemade carbon film coated grids and stained with 2% uranyl formate. Specimen was imaged with a Hitachi HT7800 electron microscope operating at 120 kV with a Xarosa CMOS camera.

In vitro nanoparticle assembly and non-assembling control preparation

Total protein concentration of purified individual nanoparticle components was determined by measuring absorbance at 280 nm using a UV/vis spectrophotometer (Agilent Cary 8454) and calculated extinction coefficients (Gasteiger et al., 2005). The assembly steps were performed at room temperature with addition in the following order: Pfs48/45-6C-I53-50A trimeric fusion protein, followed by additional buffer as needed to achieve desired final concentration, and finally I53-50B.4PT1 pentameric component or 2obx non-assembling control (in 50 mM Tris pH 8, 500 mM NaCl, 0.75% w/v CHAPS), with a molar ratio of Pfs48/45-6C-I53-50A: I53-B.4PT1 (or 2obx) of 1.1:1. All in vitro assemblies and non-assembling control preparations were incubated at 2-8°C with gentle rocking overnight before purification by SEC on a Superose 6 Increase 10/300 GL column in order to remove the residual unassembled component. Non-assembling controls were not SEC purified after preparation at the 1:1 molar ratio. Assembled nanoparticles were sterile filtered (0.22 μm) immediately prior to column application and following pooling of fractions.

Endotoxin measurements for Pfs48/45-6C-I53-50 particles and controls

Endotoxin levels in protein samples were measured using the EndoSafe Nexgen-MCS System (Charles River). Samples were diluted 1:50 or 1:100 in Endotoxin-free LAL reagent water and applied into wells of an EndoSafe LAL reagent cartridge. Charles River EndoScan-V software was used to analyze endotoxin content, automatically back-calculating for the dilution factor. Endotoxin

values were reported as EU/mL which were then converted to EU/mg based on UV/vis measurements. The threshold for samples suitable for immunization was < 50 EU/mg.

UV/vis for Pfs48/45-6C-I53-50 particles and controls

Ultraviolet-visible spectrophotometry (UV/vis) was measured using an Agilent Technologies Cary 8454. Samples were applied to a 10 mm, 50 μL quartz cell (Starna Cells, Inc.) and absorbance was measured from 180 to 1000 nm. Net absorbance at 280 nm, obtained from the measurement and single reference wavelength baseline subtraction, was used with calculated extinction coefficients and molecular weights to obtain protein concentration. The ratio of absorbance at 320/280 nm was used to determine relative aggregation levels in real-time stability study samples. Samples were diluted with respective purification/instrument blanking buffers to obtain an absorbance between 0.1 and 1.0. All data produced from the UV/vis instrument was processed in the 845x UV/visible System software

Biolayer interferometry for Pfs48/45-6C variants

Biolayer interferometry (Octet RED96, FortéBio) experiments at 25°C were conducted to determine the binding kinetics of Pfs48/45-6C variants and the TB31F Fab. Recombinant His-tagged Pfs48/45-6C was diluted into kinetics buffer (PBS, pH 7.4, 0.01% (w/v) BSA, 0.002% (v/v) Tween-20) at 15 μg/mL and immobilized onto Ni-NTA (NTA) biosensors (FortéBio). Following a 60 s baseline step, biosensors were dipped into wells containing twofold dilution series of Fab, from 500 nM to 15.6 nM (affinity measurement). Sensors were then dipped back into kinetics buffer to monitor the dissociation rate. Kinetics data were analyzed using FortéBio's Data Analysis software 9.0, and kinetic curves were fitted to a 1:1 binding model using at least four concentrations. For experiments with several replicates, the mean kinetic constants reported are the result of two or more independent experiments, and associated error is standard deviation. For RUPA-117 and TB31F Fabs binding to 6C.mAgE2 and its glycan variants, association into Fabs was performed at 10 μg/mL before being dipped back into kinetics buffer for dissociation. For quantification experiments, TB31F Fab was immobilized on an anti-human Fab CH1 biosensor (FortéBio). 6C.mAgE2 serially diluted in HEK293F supernatant to a concentration range of 0-200 μg/mL was used to generate a standard curve from the association rate. Supernatants of 6C.WT, 6C.sAgE7, 6C.mAgE1, and 6C.mAgE2 from three independent transfections were subsequently characterized. For all quantitation, the initial association slopes were used for calculations, and all analyses were done using FortéBio's Data Analysis software 9.0 and GraphPad Prism 9.

Biolayer interferometry for Pfs48/45-6C-I53-50 particles and controls

Antigenicity assays were performed and analyzed using BLI on an Octet Red 96 System (Forté Bio/Sartorius) at ambient temperature with shaking at 1000 rpm. Pfs48/45-6C-I53-50 particles and associated components with 2obx non-assembling control were diluted to 10 μg/mL in Kinetics buffer (PBS, pH 7.4, 0.01% (w/v) BSA, 0.002% (v/v) Tween-20). Samples were immobilized onto Anti-Penta-HIS (HIS1K) biosensors and reagents were applied to a black 96-well Greiner Bio-one microplate at 200 μL per well as described below. HIS1K biosensors were hydrated in Kinetics Buffer for 10 min and were then equilibrated in Kinetics buffer for 60 s. The HIS1K tips were loaded to a loading threshold of 0.7 nm and washed with kinetics buffer for 60 s. The association step was performed by dipping the HIS1K biosensors with immobilized immunogen into diluted TB31F Fab for 120 s, then dissociation was measured by inserting the biosensors back into Kinetics buffer for 240 s. The data were baseline subtracted and the plots fitted using the Pall FortéBio/Sartorius analysis software (version 12.0)

Circular dichroism

CD thermal melts were conducted at 218 nm, with a temperature ramp of 1°C per minute, from 20-105°C, a bandwidth of 2 nm, and a CD scale of 200 mdeg/1.0dOD. For full spectra analysis, samples were analyzed at 190-260 nm in a continuous manner at a rate of 50 nm/min, with a digital integration time (DIT) of 2 s, a bandwidth of 1 nm, and a CD scale of 200 mdeg/1.0dOD. All analyses were first blanked and corrected with PBS, and all samples were tested at a concentration of 0.25 mg/mL.

Dynamic light scattering for extended thermostability analysis

Isothermal DLS was conducted at 25°C, with 10 acquisitions per measurement, at a 5 s acquisition time. Of the ten acquisitions, reads that differed from the mean initial Intensity Autocorrelation were discarded, and a minimum of six acquisitions were used in data processing. Data processing was conducted using the DYNAMICS V7 software, and samples were analyzed in three ranges: 0.1-10 nm, 10-100 nm, and 100-1000 nm. All samples were tested at 1 mg/mL.

Dynamic light scattering for Pfs48/45-6C-I53-50 particles and controls

Dynamic Light Scattering (DLS) was used to measure the hydrodynamic diameter (Dh) and % Polydispersity (%Pd) of Pfs48/45-6C-I53-50 samples on an UNcle Nano-DSF (UNchained Laboratories). The sample was applied to an 8.8 mL quartz capillary cassette (UNi, UNchained Laboratories) and measured with 10 acquisitions of 5 s each, using auto-attenuation of the laser.

Electron microscopy of CoPoP liposomes

6C.mAgE2 CoPoP liposomes containing 40 μg/mL of protein and ~8.4 mg/mL total lipid were diluted five-fold and 3 μL of the sample was deposited on homemade holey gold grids (Marr et al., 2014), which were glow-discharged in air for 15 s before use. Samples

were incubated on a grid for 5 min, blotted for 3.5 s, and subsequently plunge-frozen in liquid ethane (Tivol et al., 2008) using an Automatic Plunge Freezer EM GP2 from Leica (maintained at 4°C and 100% humidity). Data collection was performed with a FEI Tecnai F20 microscope operated at 200 kV with a K2 camera (Gatan Inc). Micrographs were recorded as 15 s movies of 30 frames at 5 electrons per pixel per second and a calibrated pixel size of 1.45 Å per pixel. A defocus range between 2.0 and 3.0 μm was used for data collection.

Negative stain electron microscopy for Pfs48/45-6C-I53-50 particles and controls

Pfs48/45-6C-I53-50 nanoparticles were diluted to 0.03 mg/mL in 50 mM Tris pH 8, 150 mM NaCl, prior to application of 3 μL of sample onto freshly glow-discharged 300-mesh copper grids. The sample was incubated on the grid for 1 min before the grid was dipped in a 50 μL droplet of water and excess liquid blotted away with filter paper (Whatman). The grids were then dipped into 6 μL of 0.75% w/v uranyl formate stain. The stain was blotted off with filter paper, then the grids were dipped into another 6 μL of stain and incubated for ~70 s. Finally, the stain was blotted away, and the grids were allowed to dry for 1 min. Prepared grids were imaged in a Talos model L120C electron microscope at 45,000X magnification.

Animal immunizations with Pfs48/45-6C and Pfs48/45-6C-CoPoP

The immunogenicity of various Pfs48/45-6C constructs was assessed using two different adjuvants (Montanide ISA 720 (SEPPIC) or CPQ liposomes). To prepare the final vaccine, Montanide ISA 720 was mixed with the aqueous antigen at a ratio of 70%/30%. Conversely, CPQ liposomes were simply incubated with the constructs for 3 h at RT. Female ICR mice (Envigo order code 030) were immunized intramuscularly with 5 μg of various Pfs48/45-6C constructs in a prime/boost dose regimen at day 0 and 21; blood was collected at day 42.

Animal Immunizations of Pfs48/45-6C nanoparticles and controls

On the day of immunization, immunogens were mixed with AddaVax™ adjuvant from InvivoGen according to the manufacturer's instructions. For immunizations, 8-week female Crl:CD1(ICR) mice from Charles River Laboratories were anesthetized with isoflurane followed by intramuscular injections into the quadricep muscle. Each mouse received 0.05 mL of the immunogen in each quadricep, for a total of 0.1 mL of immunogen per mouse per immunization. Mice were injected on days 0 and 28 for a total of two immunizations. Pre-immune, day 14, and day 42 sera were collected for analysis.

ELISA and SMFA of anti-Pfs48/45 antibodies elicited by Pfs48/45-6C and Pfs48/45-6C-CoPoP at Laboratory of Malaria and Vector Research (LMVR)

The details of ELISA method have been published elsewhere (Miura et al., 2008). All ELISA were conducted using 6C.sAgE7 as a coating antigen. The ability of anti-Pfs48/45 antibodies to reduce the development of *P. falciparum* NF54 strain oocysts in the mosquito midgut was evaluated by SMFA, as described previously (Miura et al., 2013, 2016; McLeod et al., 2019). In brief, IgG was purified from whole sera using Protein G affinity chromatography, then indicated concentrations of IgG were mixed with 0.15% - 0.2% stage V gametocytemia, and then fed to 3-6-day old female *Anopheles stephensi*. All experiments were done in the presence of human complement. The mosquitoes were maintained for 8 days and then dissected to count the number of oocysts per midgut in 20 mosquitoes.

ELISA of antibodies elicited by Pfs48/45-6C nanoparticles and controls

50 μL of 2 μg/mL 6C-sAgE7 was plated onto 96-well Nunc Maxisorp (ThermoFisher) plates in TBS. Plates were incubated at 25°C for 1 h then blocked with 200 μL of 2% BSA in TBST for an additional 1 h at 25°C. Plates were washed 3 × in TBST using a plate washer (BioTek), 1:5 serial dilutions of mouse sera were made in 50 μL TBST starting at 1:100 and incubated at 25°C for 1 h. Plates were washed 3 × in TBST, then anti-mouse horseradish peroxidase-conjugated horse IgG (Cell Signaling Technology, #7076S) was diluted 1:2,000 in 2% BSA in TBST and 50 μL was added to each well and incubated at 25°C for 30 min. Plates were washed 3 × in TBST and 100 μL of TMB (SeraCare) was added to every well for 2 min at room temperature. The reaction was quenched with the addition of 100 μL of 1 N HCl. Plates were immediately read at 450 nm on a SpectraMax M5 plate reader (Molecular Devices) and data plotted and fit in Prism (GraphPad) using nonlinear four-paramater logistics sigmoidal regression, where X is log(concentration) to determine EC50 values from curve fits.

SMFA at Radboud University Medical Center (RUMC)

SMFA experiments at RUMC were conducted with *Plasmodium falciparum* NF54 gametocytes and *Anopheles stephensi* mosquitoes (Nijmegen colony), as previously described (Ponnudurai et al., 1989). Mice sera were diluted in fetal calf serum and mixed with cultured gametocytes, normal human serum with complement and red blood cells. Serum dilution factors indicate the proportion serum in the total blood meal volume. Negative controls included fetal calf serum only. Blood meals were fed to mosquitoes and 6-8 days later oocysts were counted in 20 fully-fed mosquitoes. Each sample was tested in two independent SMFA experiments. We fitted a mixed-effects negative binomial regression model to model the oocyst counts in each of the test conditions relative to a control. Log risk ratios, that show the log of the relative amount of oocysts in treatment versus control, was directly estimated in the model, and was used to calculate the TRA. Random intercepts were used to account for the correlation between mosquito observations from the same SMFA experiment.

QUANTIFICATION AND STATISTICAL ANALYSIS

To compare ELISA units or ELISA titers, the values were log-transformed, and then a Student's t-test (to compare two groups) or one-way ANOVA (for more than two groups) followed by Tukey's multiple comparison tests were utilized.

For SMFA performed at LMVR, the percent of TRA (TRA(%)), the 95% confidence interval (95%CI) and p-value from a single or multiple feeds were calculated using a zero-inflated negative binomial (ZINB) model as before (Miura et al., 2013) for SMFA conducted at LMVR. IC_{80}, IgG concentration which gives 80% TRA, was calculated from a plot where IgG concentration was transformed in a square-root scale and % TRA was transformed in a Log of mean oocyst ratio (LMR) scale, as described previously (Miura et al., 2019).

All statistical tests were performed in Prism 8 or Prism 9 (GraphPad Software, La Jolla, CA, USA) or R (version 3.5.3, The R Foundation for Statistical Computing) and p-values <0.05 are considered significant.

Immunity, Volume *55*

Supplemental information

Vaccination with a structure-based stabilized version of malarial antigen Pfs48/45 elicits ultra-potent transmission-blocking antibody responses

Brandon McLeod, Moustafa T. Mabrouk, Kazutoyo Miura, Rashmi Ravichandran, Sally Kephart, Sophia Hailemariam, Thao P. Pham, Anthony Semesi, Iga Kucharska, Prasun Kundu, Wei-Chiao Huang, Max Johnson, Alyssa Blackstone, Deleah Pettie, Michael Murphy, John C. Kraft, Elizabeth M. Leaf, Yang Jiao, Marga van de Vegte-Bolmer, Geert-Jan van Gemert, Jordache Ramjith, C. Richter King, Randall S. MacGill, Yimin Wu, Kelly K. Lee, Matthijs M. Jore, Neil P. King, Jonathan F. Lovell, and Jean-Philippe Julien

Supplementary Material

Fig S1:

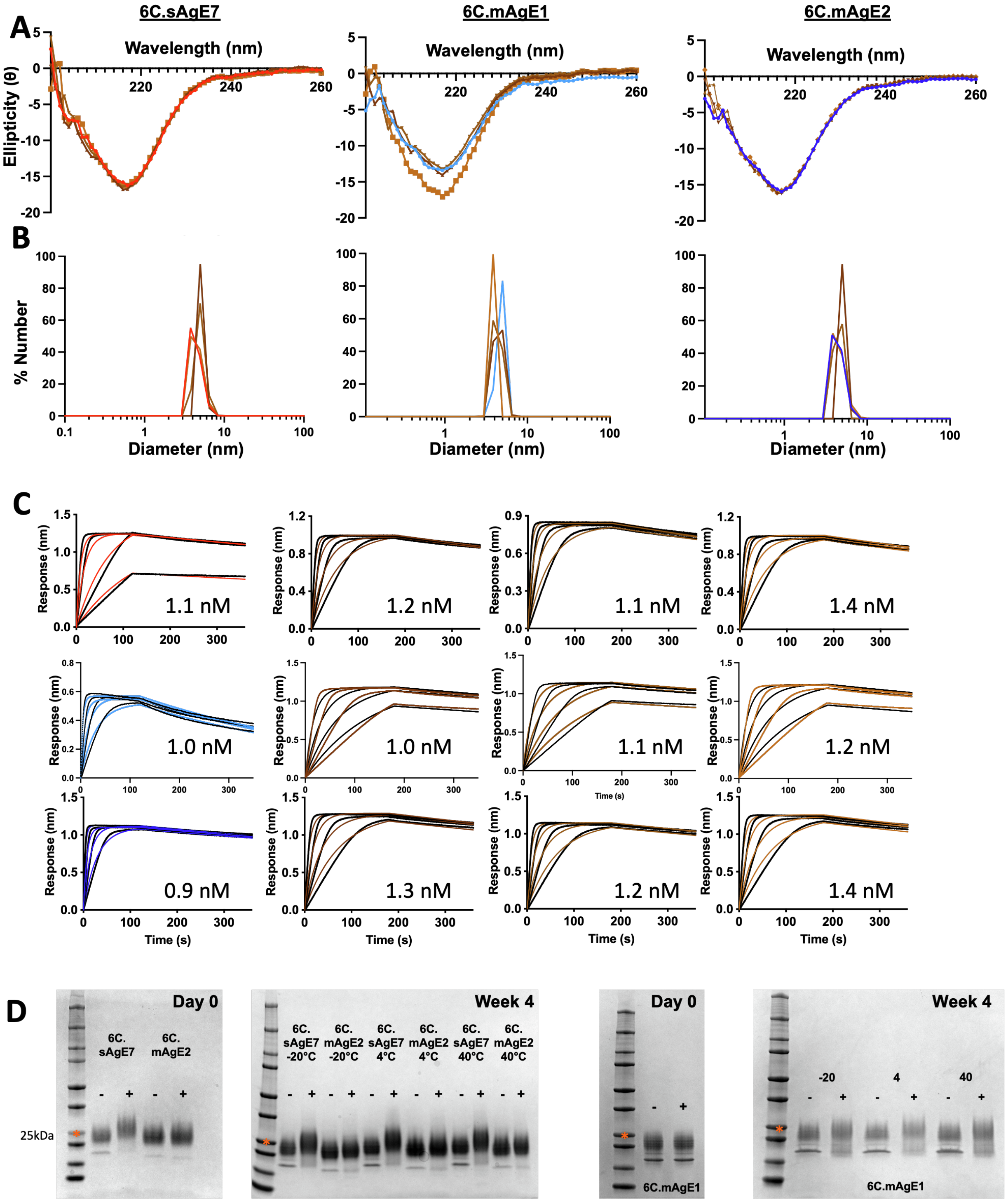

Fig S1. Accelerated thermostability assessment of 6C.sAgE7, 6C.mAgE1, and 6C.mAgE2 at Day 0 and Week 4. A) Day 0 CD wavelength scans of 6C.sAgE7 (left, red), 6C.mAgE1 (mid, pale blue), and 6C.mAgE2 (right, blue), superimposed with full CD wavelength scans from samples incubated for four weeks at -20°C (dark brown), 4°C (brown), and 40°C (light brown). **B)** Superimposition of DLS for stabilized antigens at Day 0 and Week 4, colored as in **A**. **C)** Day 0 and Week 4 representative binding curves used to calculate binding constants for 6C.sAgE7 (top), 6C.mAgE1 (middle) and 6C.mAgE2 (bottom). Affinity constant (KD) is labelled on its respective binding curve for raw data (black) and fitted data (as colored in **A**), for four serially diluted concentrations of Pfs48/45-6C antigen, from 250 - 31.3 nM) **A**. **D)** SDS-PAGE of Day 0 and Week4 samples. Symbols “-” and “+” denote which samples were run without and with reducing agent, respectively. Relevant 25 kDa band from molecular weight ladder is denoted by an orange asterisk. (related to **Fig 1**)

Fig S2:

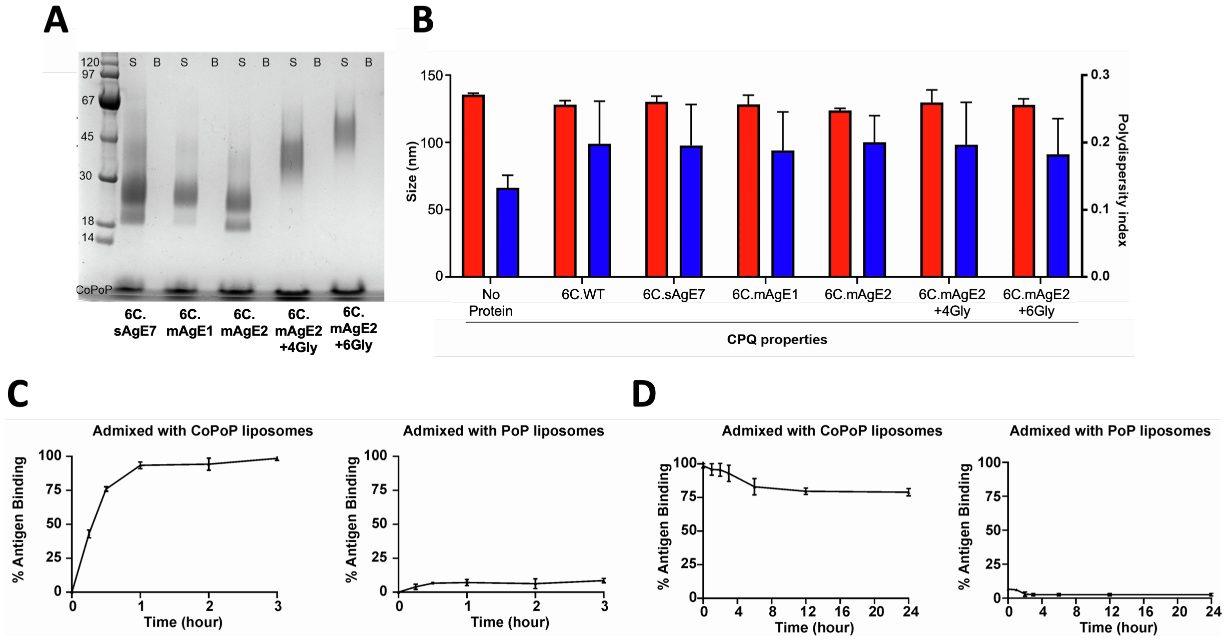

Fig S2. Particle properties of CPQ liposomes with Pfs48/45-6C variants. A) Nickel-NTA bead competition assay. Proteins were incubated with CPQ liposomes, then subjected to competition with Nickel-NTA beads. Beads were separated into the supernatant (S) and beads (B) fractions. Protein presence in the supernatant fraction reflects full binding of the protein to the liposomes. **B)** Size (red, left Y-axis) and polydispersity (blue, right Y-axis) of liposomes displaying the various indicated Pfs48/45-6C constructs plotted as mean, with error as standard deviation. **C)** Fluorescently labeled Pfs48/45-6C was incubated with CPQ liposomes or similar liposomes lacking cobalt within the porphyrin-phospholipid (PoP) as indicated to determine binding kinetics in PBS. Upon binding to liposomes, energy transfer induces a decrease in fluorescence reflecting antigen binding. Data is plotted as mean, with standard deviation as error. **D)** Following complexation, stability in 20% serum over time at 37 °C. Data is plotted as mean, with standard deviation as error. (related to **Fig 2**)

Fig S3:

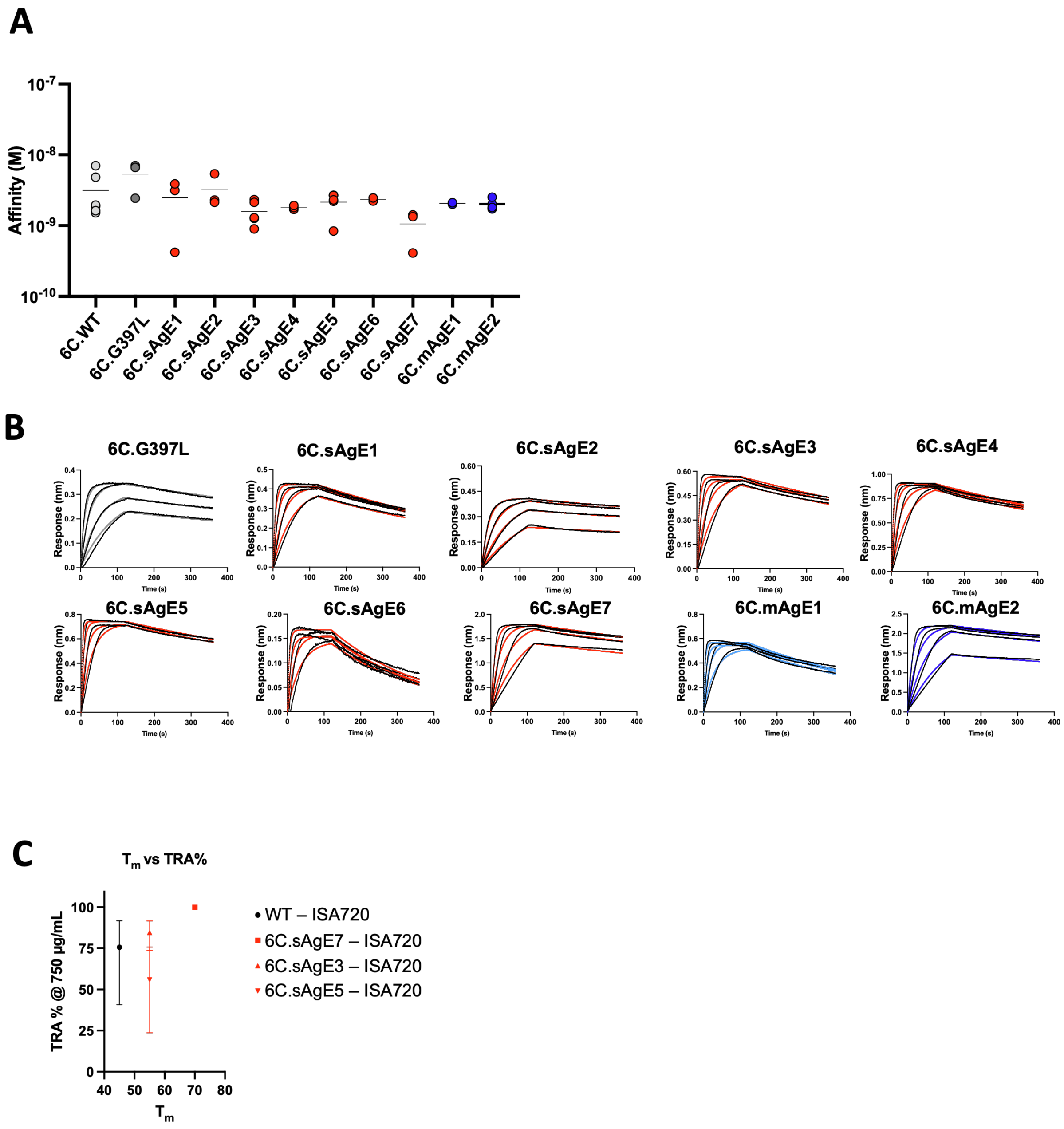

Fig S3. Biophysical characterization of Pfs48/45-6C stability mutations. A) Affinity of TB31F Fab binding to Pfs48/45-6C variants as measured by BLI. Symbols are representative of individual measurements, with black horizontal bar indicating the mean. **B)** Representative curves of

TB31F Fab binding to Pfs48/45-6C variants as measured by BLI. Curves are presented as raw data (black), and fitted data (as coloured in **A**), for four serially diluted concentrations of 6C antigen, from 250 - 31.3 nM) **C)** SMFA for IgG purified from mice immunized with ISA720-adjuvanted antigens were tested at 750 μg/mL, and plotted as a function of thermostability (T_m). Symbol represents the best-estimate TRA(%) of the two independent feeds, and error bars are 95% CI. (related to **Figs 1-3**)

Fig S4:

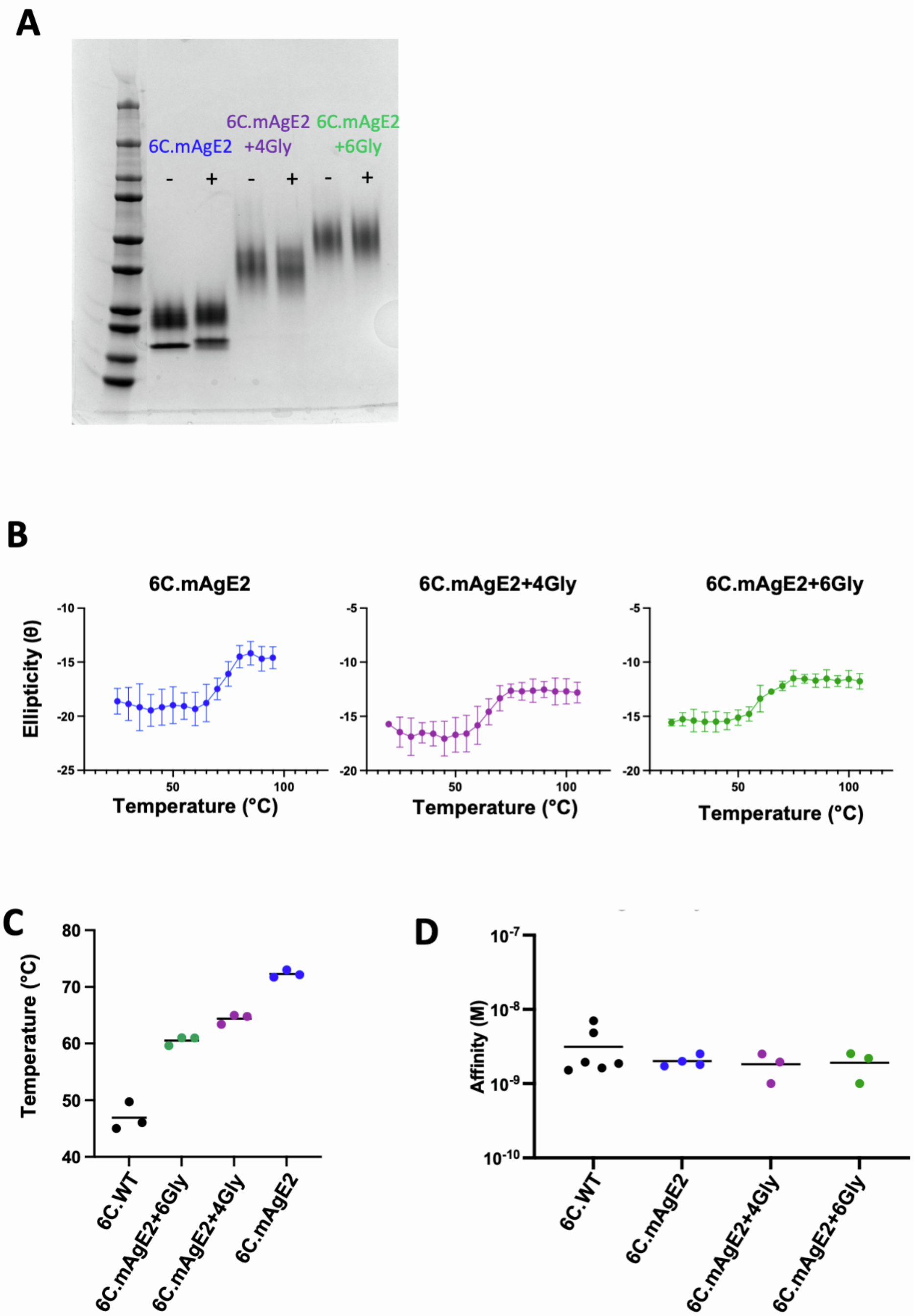
A
6C.mAgE2
6C.mAgE2 +4Gly
6C.mAgE2 +6Gly
- + - + - +
B
6C.mAgE2
6C.mAgE2+4Gly
6C.mAgE2+6Gly
Ellipticity (θ)
Temperature (°C)
C
Temperature (°C)
6C.WT
6C.mAgE2+6Gly
6C.mAgE2+4Gly
6C.mAgE2
D
Affinity (M)
6C.WT
6C.mAgE2
6C.mAgE2+4Gly
6C.mAgE2+6Gly

Fig S4. Characterization of hyperglycosylated Pfs48/45-6C variants. A) SDS-PAGE of 6C.mAgE2 hyperglycosylated variants in comparison to 6C.mAgE2. **B)** CD melting curves for 6C.mAgE2 and hyperglycosylated variants. Data points represent mean of three independent replicates and error bars are plotted as standard deviation. **C)** Thermostability of 6C.WT, 6C.mAgE2 and hyperglycosylated variants, as determined by CD melting curves in **B**. Data is plotted with individual replicates, with mean as black bar. **D)** Affinity measurements of TB31F Fab to 6C.WT, 6C.mAgE2 and hyperglycosylated variants via BLI. Data are plotted as independent replicates, with the black bar representing the mean. (related to **Fig 4**)

Fig S5:

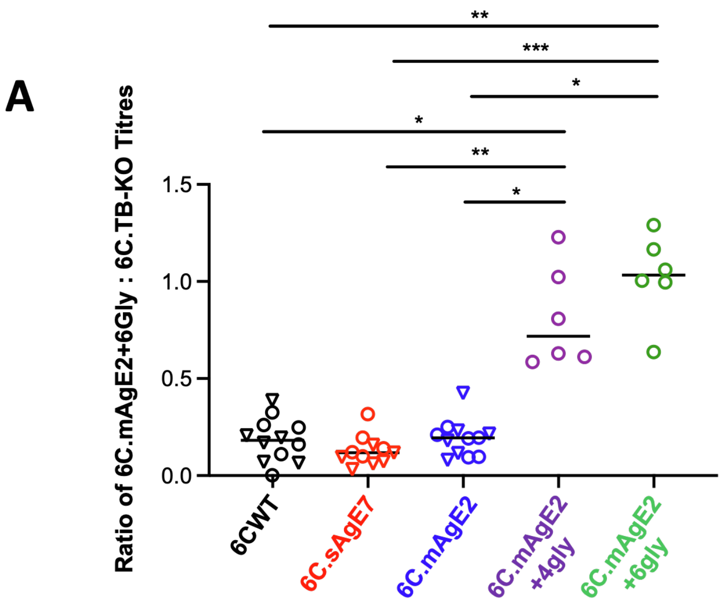

Fig S5. Characterization of immune response elicited by hyperglycosylated Pfs48/45-6C variants. Ratio of ELISA titres derived by dividing reactogenicity to 6C.mAgE2+6gly over reactogenicity to a Pfs48/45-6C TB31F epitope-knockout construct. Data is plotted as responses from individual mice, with the horizontal black bar representing the mean. *, **, and *** indicate p values of ≤0.05, ≤0.01, and ≤0.001, respectively as determined by one-way ANOVA. (related to **Fig 4**)

Fig S6:

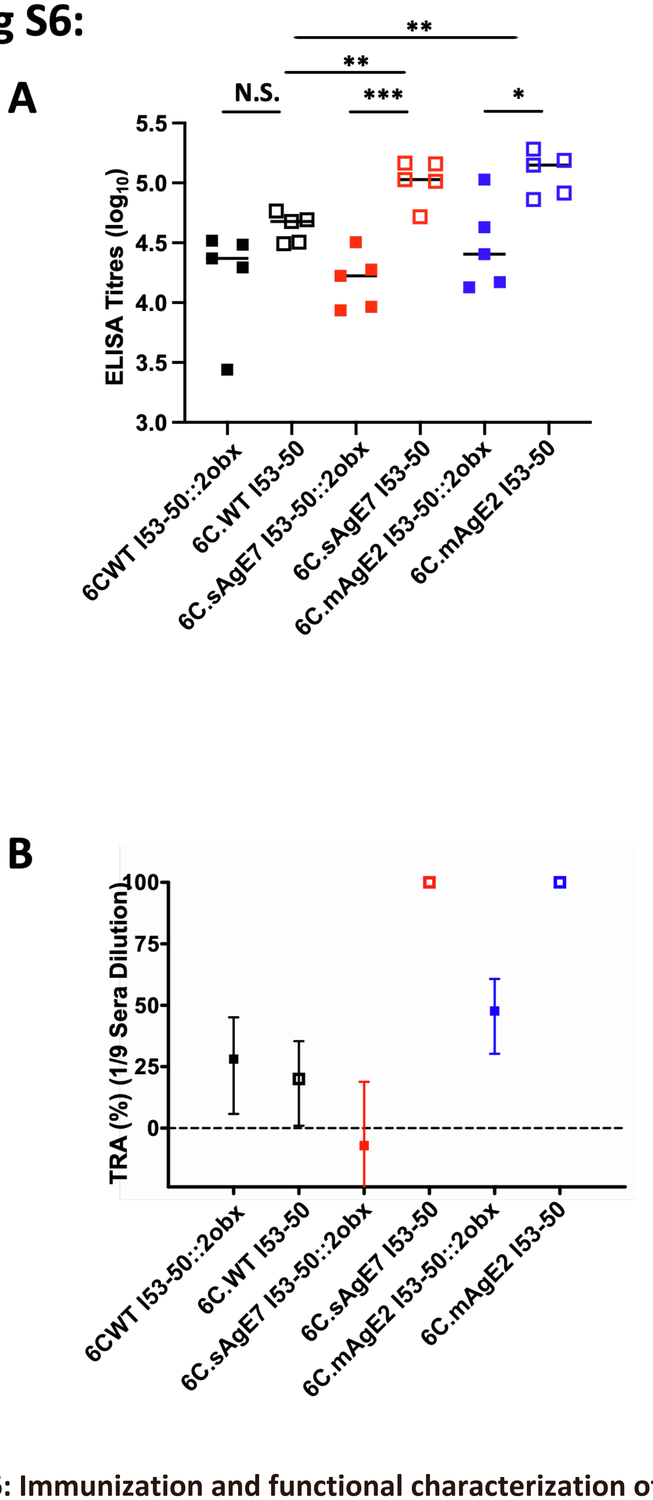

Fig S6: Immunization and functional characterization of Pfs48/45-6C stabilized variants on I53-50 protein nanocages and controls. A) 6C.sAgE7 titres elicited by stabilized Pfs48/45-6C variants, as non-assembling mixtures of Pfs48/45-6C-I53-50A trimers and 2obx pentamers

(filled squares) or icosahedral I53-50 protein nanocages (open squares), measured by ELISA. Data is plotted as individual mice, with the horizontal black bars representing the mean. n.s. non-significant ($p>0.05$); *, **, and *** indicate p values of ≤0.05, ≤0.01, and ≤0.001, respectively as determined by one-way ANOVA for log-transformed ELISA titres. **B)** SMFA with 1:9 diluted pooled sera derived from mice immunized with stabilized Pfs48/45-6C variants at a 1:9 dilution for non-assembling controls (filled circles) or icosahedral particles (open circles), plotted as mean with error as standard deviation. (related to **Fig 5**)

Supplementary table 1: Data collection and refinement statistics for the 6C.mAgE1-RUPA-47 Fab-RUPA-117 Fab complex. (related to **Fig 3**)

	6C.mAgE1-RUPA-47 Fab-RUPA-117 Fab
**Wavelength (Å)**	1.033190
**Space group**	$P2_12_12_1$
**Cell dimensions**	
a, b, c (Å)	73.7 127.2 132.7
α, β, γ **(°)**	90 90 90
**Resolution (Å)**	29.43 - 2.18 (2.21 - 2.18)
**No. molecules in ASU**	1
**No. unique observations**	65,804 (2596)
**Multiplicity**	12.6 (7.8)
**R_{merge} (%)**	20.5 (73.3)
**R_{pim} (%)**	5.9 (28.1)
**<I/sigma I>**	8.7 (1.5)
**$CC_{1/2}$**	99.5 (60.3)
**Completeness (%)**	99.9 (100)
**Refinement Statistics**	
**Non-hydrogen atoms**	8,338
**Macromolecule**	7,730
**Water**	594
**Hetero atom**	14
**R_{factor}/ R_{free}**	17.8/ 22.8
**Rms deviations from ideality**	
**Bond lengths (Å)**	0.003
**Bond angle (°)**	0.66
**Ramachandran plot**	
**Favoured regions (%)**	97.5
**Allowed regions (%)**	2.5
**Outliers (%)**	0.1
**B-factors ($Å^2$)**	
**Wilson B-factor**	31.0
**Average B-factors**	33.2
**Average macromolecule**	32.9
**Average hetero atom**	63.3
**Average water**	36.4

Supplementary table 2: Conditions for HDX-MS experiment. (related to **Fig 3**)

Dataset	6C.sAgE7	6C.mAgE2
HDX reaction details	85% D2O buffer, pH* 7.659, labeled at RT. Quenched at pH 2.55 in 200mM TCEP, 0.2%FA	85% D2O buffer, pH* 7.659, labeled at RT. Quenched at pH 2.55 in 200mM TCEP, 0.2%FA
HDX time course	3 s, 1 min, 30 min, 5 h	3 s, 1 min, 30 min, 5 h
HDX controls	PPPI, PPPF	PPPI, PPPF
Back-exchange	37.1 +/- 7.8%	37.0 +/- 8.7%
Number of peptides	32	39
Sequence coverage	87%	88%
Average peptide length/redundancy	11.4 residue length/2.8 redundancy	11.3 residue length/ 3.25 redundancy
Replicates (biological or technical)	two technical replicates	two technical replicates
Repeatability	stddev of 1.2% across technical replicates	stddev of 1.2% across technical replicates

Supplementary Table 3: SMFA data for Fig 2C and 2D

Feed #	Animal study #	Immunogen	Adjuvant	IgG Conc. [µg/mL]	Mean oocyst	TRA (%)	p-value[a]
#296	*Control IgG*			*750*	*66*		
	UB19	6C.sAgE3	CPQ	750	42	37	0.241
	UB19	6C.sAgE3	ISA	750	22	67	0.005
	UB19	6C.sAgE5	CPQ	750	42	36	0.277
	UB19	6C.sAgE5	ISA	750	27	60	0.018
	UB19	6C.sAgE7	CPQ	750	0	100	0.001
	UB19	6C.sAgE7	ISA	750	0	100	0.001
#304	*Control IgG*			*750*	*57*		
	UB19	6C.sAgE7	CPQ	750	0	100	0.001
	UB19	6C.sAgE7	ISA	750	0	100	0.001
#312	*Control IgG*			*750*	*15*		
	UB21	6C.WT	CPQ	750	3	78	0.002
	UB21	6C.WT	ISA	750	4	76	0.003
#321	*Control IgG*			*750*	*16*		
	UB19	6C.sAgE3	CPQ	750	6	64	0.019
	UB19	6C.sAgE3	ISA	750	1	93	0.001
	UB19	6C.sAgE5	CPQ	750	5	70	0.005
	UB19	6C.sAgE5	ISA	750	8	52	0.089

[a] The p-values were calculated using a zero-inflated negative binomial (ZINB) model (Miura *et al.*, 2016).

Supplementary Table 4: SMFA data for Fig 2E

Feed #	Animal study #	Immunogen	Adjuvant	Anti-6C.sAgE7 ELISA units	Mean oocyst	TRA (%)	p-value
#296	*Control IgG*				*66*		
	UB19	6C.sAgE7	CPQ	21,455	0	100	0.001
	UB19	6C.sAgE7	ISA	7,410	0	100	0.001
#304	*Control IgG*				*57*		
	UB19	6C.sAgE7	CPQ	21,455	0	100	0.001
	UB19	6C.sAgE7	ISA	7,410	0	100	0.001
#312	*Control IgG*				*15*		
	UB21	6C.sAgE7	CPQ	21,455	0	100	0.001
	UB21	6C.sAgE7	ISA	7,410	0	100	0.001
#315	*Control IgG*				*54*		
	UB21	6C.sAgE7	CPQ	1,345	8	85	0.001
				2,689	1	97	0.001
				5,378	0	100	0.001
				10,728	0	100	0.001
	UB21	6C.sAgE7	ISA	464	39	28	0.389
				929	47	12	0.726
				1,857	26	52	0.078
				3,705	5	92	0.001
#317	*Control IgG*				*14*		
	UB21	6C.sAgE7	CPQ	2,689	0	99	0.001
				5,378	0	100	0.001
				10,728	0	100	0.001
#321	*Control IgG*				16		
	UB21	6C.sAgE7	CPQ	671	5	65	0.015
				1,341	2	90	0.001
#325	*Control IgG*				*9*		
	UB21	6C.sAgE7	CPQ	671	8	9	0.799

Supplementary Table 5: SMFA data for Fig 4C

Feed #	Animal study #	Immunogen	Adjuvant	IgG Conc. [μg/mL]	Mean oocyst	TRA (%)	p-value
#317	*Control IgG*				*14*		
	UB22	6C.mAgE2	CPQ	375	0	100	0.001
#318	*Control IgG*				*6*		
	UB22	6C.mAgE2	CPQ	375	0	100	0.001
	UB22	6C.mAgE2+4gly	CPQ	375	1	90	0.001
	UB22	6C.mAgE2+6gly	CPQ	375	9	-38	0.423
#321	*Control IgG*				*16*		
	UB22	6C.mAgE2+4gly	CPQ	375	3	83	0.001
	UB22	6C.mAgE2+6gly	CPQ	375	4	72	0.004

Supplementary Table 6: SMFA data for Fig 5C

Feed #	Animal study #	Immunogen	Adjuvant	IgG Conc. [µg/mL]	Mean oocyst	TRA (%)	p-value
#296	*Control IgG*				*66*		
	UB19	6C.sAgE7	CPQ	750	0	100	0.001
#304	*Control IgG*				*57*		
	UB19	6C.sAgE7	CPQ	750	0	100	0.001
#312	*Control IgG*				*15*		
	UB21	6C.WT	CPQ	750	3	78	0.002
	UB21	6C.sAgE7	CPQ	750	0	100	0.001
	UB21	6C.mAgE1	CPQ	750	0	100	0.001
	UB21	6C.mAgE2	CPQ	750	0	100	0.001
#315	*Control IgG*				*54*		
	UB21	6C.sAgE7	CPQ	47	8	85	0.001
			CPQ	94	1	97	0.001
			CPQ	188	0	100	0.001
			CPQ	375	0	100	0.001
	UB21	6C.mAgE2	CPQ	47	34	38	0.224
			CPQ	94	22	59	0.036
			CPQ	188	5	91	0.001
			CPQ	375	0	99	0.001
#317	*Control IgG*				*14*		
	UB21	6C.mAgE1	CPQ	94	0	100	0.001
			CPQ	188	0	100	0.001
			CPQ	375	0	100	0.001
	UB21	6C.mAgE2	CPQ	94	10	25	0.479
			CPQ	188	1	92	0.001
			CPQ	375	0	100	0.001
	UB21	6C.sAgE7	CPQ	94	0	99	0.001
				188	0	100	0.001
				375	0	100	0.001
	UB22	6C.mAgE2	CPQ	94	1	96	0.001
				188	0	100	0.001
				375	0	100	0.001
	UB22	6C.sAgE7	CPQ	94	0	100	0.001
			CPQ	188	0	100	0.001
			CPQ	375	0	100	0.001
#318	*Control IgG*				*6*		
	UB22	6C.WT	CPQ	375	4	34	0.287
	UB22	6C.sAgE7	CPQ	375	0	100	0.001
	UB22	6C.mAgE2	CPQ	375	0	100	0.001

Supplementary Table 7: SMFA data for Fig 5C (continued)

Feed #	Animal study #	Immunogen	Adjuvant	IgG Conc. [μg/mL]	Mean oocyst	TRA (%)	p-value
#321	*Control IgG*				*16*		
	UB21	6C.mAgE1	CPQ	23	4	72	0.004
			CPQ	47	3	81	0.001
			CPQ	94	1	91	0.001
	UB21	6C.sAgE7	CPQ	23	5	65	0.015
			CPQ	47	2	90	0.001
	UB22	6C.mAgE2	CPQ	23	2	86	0.001
			CPQ	23	7	56	0.049
			CPQ	47	4	76	0.001
			CPQ	94	2	89	0.001
			CPQ	188	1	96	0.001
	UB22	6C.sAgE7	CPQ	47	1	91	0.001
	UB21	6C.mAgE1	CPQ	23	4	72	0.004
			CPQ	47	3	81	0.001
#325	*Control IgG*				*9*		
	UB21	6C.mAgE1	CPQ	23	5	48	0.119
			CPQ	47	0	95	0.001
			CPQ	94	0	100	0.001
			CPQ	188	0	100	0.001
			CPQ	375	0	100	0.001
	UB21	6C.sAgE7	CPQ	23	8	9	0.799
	UB22	6C.mAgE2	CPQ	23	6	37	0.276
			CPQ	47	3	69	0.015
	UB22	6C.sAgE7	CPQ	23	6	37	0.228
			CPQ	47	3	64	0.014
			CPQ	94	1	94	0.001
			CPQ	188	0	100	0.001
	UB22	6C.WT	CPQ	375	4	73	0.006
#332	*Control IgG*				*100*		
	UB21	6C.WT	CPQ	375	47	53	0.051
				750	52	48	0.093
	UB22	6C.WT	CPQ	750	65	35	0.264

Supplementary Table 8: SMFA data for Fig S3C

Feed #	Animal study #	Immunogen	Adjuvant	IgG Conc. [μg/mL]	Mean oocyst	TRA (%)	p-value
#296	*Control IgG*				*66*		
	UB19	6C.sAgE3	CPQ	750	22	67	0.005
	UB19	6C.sAgE5		750	27	60	0.018
	UB19	6C.sAgE7		750	0	100	0.001
#304	*Control IgG*				*57*		
	UB19	6C.sAgE7	CPQ	750	0	100	0.001
#321	*Control IgG*				*16*		
	UB19	6C.sAgE3	CPQ	750	1	93	0.001
	UB19	6C.sAgE5	CPQ	750	8	52	0.089

Supplementary Table 9: SMFA data for Fig S6B and Fig 5G

Feed #	Immunogen	Adjuvant	Sera dilution	Mean oocyst	TRA (%)
#192-2021	*FCS control*			*8.6*	
	6C.WT I53-50::2obx	AddaVax	9	5.2	39.8
	6C.sAgE7 I53-50::2obx	AddaVax	9	8.0	7.0
	6C.mAgE2 I53-50::2obx	AddaVax	9	3.8	55.6
	6C.WT I53-50	AddaVax	9	4.2	51.5
	6C.WT I53-50	AddaVax	27	9.6	-12.3
	6C.sAgE7 I53-50	AddaVax	9	0	100
	6C.sAgE7 I53-50	AddaVax	27	0	100
	6C.mAgE2 I53-50	AddaVax	9	0	100
	6C.mAgE2 I53-50	AddaVax	27	0	100
	6C.mAgE2 HpFerritin	AddaVax	9	0	100
	6C.mAgE2 HpFerritin	AddaVax	27	0	100
#013-2022	*FCS control*			*16.4*	
	6C.WT I53-50::2obx	AddaVax	9	13.7	16.8
	6C.sAgE7 I53-50::2obx	AddaVax	9	20.0	-21.6
	6C.mAgE2 I53-50::2obx	AddaVax	9	9.8	40.2
	6C.WT I53-50	AddaVax	9	13.6	17.4
	6C.sAgE7 I53-50	AddaVax	9	0	100
	6C.sAgE7 I53-50	AddaVax	27	0.1	99.7
	6C.sAgE7 I53-50	AddaVax	81	0.8	95.4
	6C.sAgE7 I53-50	AddaVax	243	8.0	51.5
	6C.mAgE2 I53-50	AddaVax	9	0	100
	6C.mAgE2 I53-50	AddaVax	27	0	100
	6C.mAgE2 I53-50	AddaVax	81	0	100
	6C.mAgE2 I53-50	AddaVax	243	2.0	87.8
	6C.mAgE2 HpFerritin	AddaVax	9	0	100
	6C.mAgE2 HpFerritin	AddaVax	27	0	100
	6C.mAgE2 HpFerritin	AddaVax	81	0.8	95.4
	6C.mAgE2 HpFerritin	AddaVax	243	6.5	60.7

#024-2022	*FCS control*			*14.1*	
	6C.WT I53-50	AddaVax	9	15.6	-10.5
	6C.WT I53-50	AddaVax	27	18.2	-29.0
	6C.sAgE7 I53-50	AddaVax	81	3.4	76.2
	6C.sAgE7 I53-50	AddaVax	243	13.9	1.6
	6C.mAgE2 I53-50	AddaVax	81	1	92.9
	6C.mAgE2 I53-50	AddaVax	243	7.3	48.1
	6C.mAgE2 HpFerritin	AddaVax	81	1.5	86.7
	6C.mAgE2 HpFerritin	AddaVax	243	13.7	3.0

Supplementary Table 10: Calculated TRA estimates for Fig S6B and Fig 5G

Immunogen	Adjuvant	Sera dilution	Calculated % TRA (95% confidence intervals)	p-value
6C.WT I53-50::2obx	AddaVax	9	28.1 (5.7-45.1)	0.009
6C.sAgE7 I53-50::2obx	AddaVax	9	-7.2 (-41.5-18.8)	0.688
6C.mAgE2 I53-50::2obx	AddaVax	9	47.7 (30.2-60.7)	0.000
6C.WT I53-50	AddaVax	9	20.0 (1.0-35.4)	0.020
6C.WT I53-50	AddaVax	27	-19.5 (-53.7-7.0)	0.918
6C.sAgE7 I53-50	AddaVax	9	100 (100-100)	0.000
6C.sAgE7 I53-50	AddaVax	27	99.8 (98.6-100)	0.000
6C.sAgE7 I53-50	AddaVax	81	86.7 (81.0-90.6)	0.000
6C.sAgE7 I53-50	AddaVax	243	27.6 (5.2-44.7)	0.010
6C.mAgE2 I53-50	AddaVax	9	100 (100-100)	0.000
6C.mAgE2 I53-50	AddaVax	27	100 (100-100)	0.000
6C.mAgE2 I53-50	AddaVax	81	96.7 (94.5-98.0)	0.000
6C.mAgE2 I53-50	AddaVax	243	70.3 (59.8-78.0)	0.000
6C.mAgE2 HpFerritin	AddaVax	9	100 (100-100)	0.000
6C.mAgE2 HpFerritin	AddaVax	27	100 (100-100)	0.000
6C.mAgE2 HpFerritin	AddaVax	81	92.3 (88.5-94.9)	0.000
6C.mAgE2 HpFerritin	AddaVax	243	34.1 (14.5-49.3)	0.001
